# Rare Copy Number Variants Are a Common Cause of Short Stature

**DOI:** 10.1371/journal.pgen.1003365

**Published:** 2013-03-14

**Authors:** Diana Zahnleiter, Steffen Uebe, Arif B. Ekici, Juliane Hoyer, Antje Wiesener, Dagmar Wieczorek, Erdmute Kunstmann, André Reis, Helmuth-Guenther Doerr, Anita Rauch, Christian T. Thiel

**Affiliations:** 1 Institute of Human Genetics, Friedrich-Alexander University Erlangen-Nuremberg, Erlangen, Germany; 2 Institut für Humangenetik, Universitätsklinikum Essen, University of Duisburg-Essen, Essen, Germany; 3 Institute of Human Genetics, University of Wuerzburg, Wuerzburg, Germany; 4 Department of Pediatrics and Adolescent Medicine, Friedrich-Alexander University Erlangen-Nuremberg, Erlangen, Germany; 5 Institute of Medical Genetics, University of Zurich, Schwerzenbach-Zurich, Switzerland; Georgia Institute of Technology, United States of America

## Abstract

Human growth has an estimated heritability of about 80%–90%. Nevertheless, the underlying cause of shortness of stature remains unknown in the majority of individuals. Genome-wide association studies (GWAS) showed that both common single nucleotide polymorphisms and copy number variants (CNVs) contribute to height variation under a polygenic model, although explaining only a small fraction of overall genetic variability in the general population. Under the hypothesis that severe forms of growth retardation might also be caused by major gene effects, we searched for rare CNVs in 200 families, 92 sporadic and 108 familial, with idiopathic short stature compared to 820 control individuals. Although similar in number, patients had overall significantly larger CNVs (p-value<1×10^−7^). In a gene-based analysis of all non-polymorphic CNVs>50 kb for gene function, tissue expression, and murine knock-out phenotypes, we identified 10 duplications and 10 deletions ranging in size from 109 kb to 14 Mb, of which 7 were *de novo* (p<0.03) and 13 inherited from the likewise affected parent but absent in controls. Patients with these likely disease causing 20 CNVs were smaller than the remaining group (p<0.01). Eleven (55%) of these CNVs either overlapped with known microaberration syndromes associated with short stature or contained GWAS loci for height. Haploinsufficiency (HI) score and further expression profiling suggested dosage sensitivity of major growth-related genes at these loci. Overall 10% of patients carried a disease-causing CNV indicating that, like in neurodevelopmental disorders, rare CNVs are a frequent cause of severe growth retardation.

## Author Summary

With a frequency of 3%, shortness of stature is a common medical concern. Although family studies have clearly shown that gene defects play a pivotal role in the development of short stature, the underlying genetic variants involved remain unknown in about 80% of cases. In contrast to recent studies which aimed at the identification of common genetic variants to explain minor differences in the height variation in the general population, we targeted rare genomic variants where we expected a major gene effect on growth. By examining 200 patients clinically evaluated for short stature, we show that rare structural chromosomal aberrations (CNVs) are associated with shortness of stature in 10% of the cases. The identified CNVs were either de novo or segregated with short stature in the families and include genes that are functionally involved in growth regulation in humans or mice. We furthermore demonstrate an overlap of these CNVs with known microdeletion syndromes. Interestingly, 3 CNVs contain positions of common variants and confirm the localization of major growth-related genes. These findings are particularly important for identification of biological pathways leading to short stature, but also for further therapeutic approaches.

## Introduction

Human growth is a highly complex and multifactorial trait, with an estimated heritability of about 80–90% [1]. Since 3% of the general population present with a body height below -2 SD scores (SDS), shortness of stature is one of the common medical concerns in childhood. Uncovering the genetic basis of short stature is not only important for clinical diagnosis, prognosis and genetic counseling of affected individuals and their families, but is also a prerequisite for future development of therapeutic approaches.

In clinical terms, short stature is divided into non-syndromic and syndromic forms, the latter affecting additional distinctive organ systems like brain and heart. In both forms growth retardation can either be of intrauterine or postnatal onset. A disproportion between the limbs and trunk is usually attributed to dysfunctional bone maturation or differentiation. Elucidation of the genetic basis of skeletal dysplasias has highlighted central defects in extracellular proteins, metabolic pathways, signal transduction mechanisms, core proteins, oncogenes and genes processing RNA and DNA as underlying mechanisms of growth [2]–[4]. However, skeletal dysplasias are rare [5], and the most common known causes of short stature are a dysfunctional growth hormone pathway, deficiency of the transcription factor SHOX and Ullrich-Turner syndrome in women [6]–[8]. After excluding these known defects the underlying cause remains unknown in approximately 80% of patients [8]–[10].

Many studies of copy number variants (CNVs) in patients with neuropsychiatric conditions or multiple congenital anomalies showed that *de novo* or inherited CNVs are pathogenic in up to 20% of patients [11], [12]. With an intermediate length of 1 kb to several Mb they include both duplications and deletions and can affect single exons, one or several genes as well as regulatory sequences. Unraveling pathogenic CNVs by molecular karyotyping also provided new opportunities to identify the genetic basis of several monogenic human diseases [13]–[16].

In this report we present the results of copy number detection in a study group of 200 patients with idiopathic short stature. Based on our hypothesis of rare variants involved in the frequent phenotype of growth retardation, we provide evidence of underlying CNVs in 10% of these patients in a gene based approach. These CNVs encompass known microaberration syndrome regions as well as *de novo* or inherited regions not yet associated with short stature but containing GWAS loci for height.

## Results/Discussion

We now recruited a group of 200 individuals and their families with idiopathic short stature seen in the genetic clinic of the Institute of Human Genetics at the University of Erlangen-Nuremberg to identify yet unknown genetic factors of growth retardation. Height adjusted SD scores were calculated on basis of the Prader Growth charts [17]. We included patients with a height standard deviation score (SDS) of below −2 based on population data or who are significantly below the expected target height for their family. Common causes of short stature such as growth hormone deficiency, Ullrich-Turner syndrome, and SHOX deficiency were excluded where applicable. All patients underwent detailed clinical and dysmorphological evaluation by one of the authors (C.T.T.) and were classified as non-specific for any known genetic aberration. Our study group included 131 patients with isolated short stature (Table 1). 69 individuals presented with additional features such as malformations or a dysmorphic facial gestalt. The mean height SDS was −2.75. 52 individuals showed severe growth retardation of prenatal onset. Patients with significant body disproportions indicating skeletal dysplasias were considered a distinct aetiological group and were not included in this study [4], [18]. Only patients with disproportionate short stature but without radiographic signs suggestive of skeletal dysplasias were retained. A borderline IQ in the range of learning disability was observed in 3%. As these individuals received regular education no specific developmental assessment was available. A control cohort used to exclude common copy number polymorphisms consisted of 820 individuals originating from the same Central European region with either exfoliation syndrome or psoriatic arthritis [19], both late onset disorders not associated with short stature. Copy number variants as a cause of these disorders were foremost excluded and not reported in the literature.

**Table 1:** Overview of the phenotypic characteristics of the patient group.

Feature	Patients (%)
Total	200
Male/Female	82 (41)/118 (59)
Mean standard deviation score (SDS) at birth	−1.1
Mean standard deviation score (SDS)	−2.75
Proportionate/disproportionate	170 (85)/30 (15)
Syndromic/isolated short stature	69 (34.5)/131 (65.5)
Primordial growth retardation yes/no	52 (26)/148 (74)
Intellectual status normal/mild learning disability	148 (74)/52 (26)

**Figure 1:**
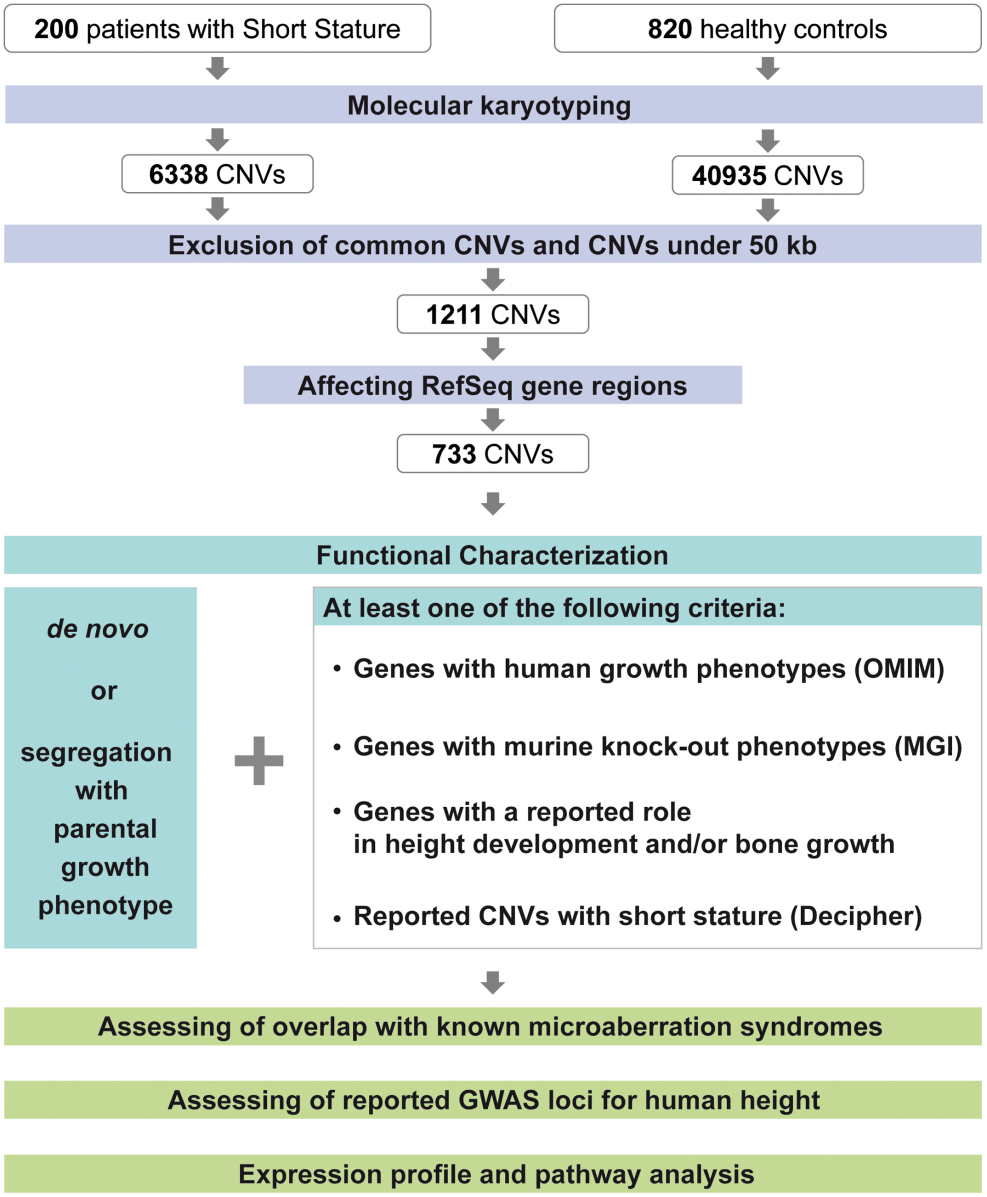
CNV discovery and characterization. Molecular karyotyping was performed for 200 patients with short stature after thorough clinical evaluation and for 820 healthy control samples. Exclusion of common variants using the control samples and scoring CNVs above 50 kb resulted in an approx. reduction of 80% of the CNVs identified in the patient samples. 60.5% of these CNVs affect reference sequence gene regions. Functional characterization includes segregation analysis using parental arrays and/or MLPA as well as gene and CNV based evaluation.

**Figure 2:**
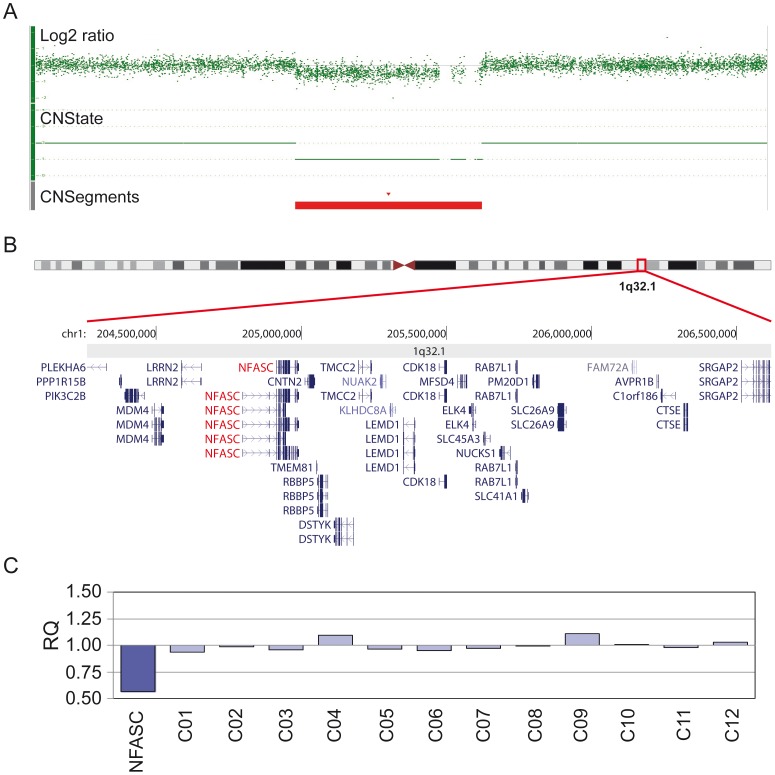
Molecular karyotyping and MLPA confirmation of identified loci. (A) Example representation of the copy number analysis of patient 1 using the Affymetrix Genotyping Console 3.0.2 software. The red bar shows the 2.2 Mb deletion region (CNSegments). (B) Graphical presentation of the deletion region including 33 candidate genes (modified from UCSC genome browser). (C) MLPA confirmation with a probe in the *NFASC* gene region. A relative quantity value (RQ) below 0.75 was considered as confirmation of a deletion, above 1.25 as confirmation of a duplication. Detailed data for the remaining patients is presented in the supporting information.

**Figure 3:**
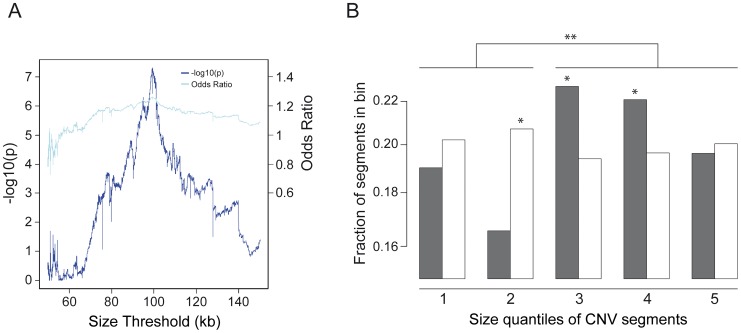
Higher incidence of CNVs with a length of above 100 kb in affected individuals. (A) Presentation of the Odds Ratio (light blue) and –log10(p-value) (dark blue) for determination of the size threshold of the number of CNVs in patients vs. controls. The Odds Ratio and the –log10(p-value) confirms a CNV size cut-off at 99.2 kb (OR 1.26 and p-value 4.98×10^−8^). (B) Fraction of copy numbers segments in cases (grey) vs. control (white) quintiles (* p<0.005). Quintile borders were Q1: 68.3 kb, Q2: 99.3 kb, Q3 149.6 kb, Q4 298.1 kb, and Q5: 72,571.3 kb. The y axis presents the fraction of CNVs inside the corresponding quintile bin. Significance levels are calculated using Fisher's exact test. The figure shows a shift towards segments above 100 kb in patients (** p-value 1.188×10^−7^).

Molecular karyotyping of the 200 patient and the 820 control samples was performed using Genome-Wide Human SNP 6.0 or CytoScan HD arrays (Figure 1). All samples met in-house quality criteria. Overall, we detected 6,338 copy number changes with an average of 32 aberrations per affected individual (Figure 2). When comparing the size range of all observed CNVs in patients (6,338 CNVs) and controls (40,935 CNVs) we determined a size threshold at 99.2 kb (Figure 3A) and found a higher incidence of CNVs with a length of above 100 kb in affected individuals (p-value 1.188×10^−7^) (Figure 3B). To test for effects of common variants we performed a genome wide CNV association analysis calculating a permutation-based p-value across the CNVs of all individuals. As expected regarding the small cohort size, genome wide association at SNP level to the 20 loci where we identified rare variants was excluded (Figure S1).

In an attempt to further investigate the variants under a “frequent disease - rare variant” – hypothesis to identify major gene effects we excluded frequent copy number polymorphisms by screening against 40,935 CNVs of the 820 control individuals (Figure 1). As we suspected low penetrance alleles to be also present in the control group, we only excluded CNVs with an overlap in CNV size of 95% in more than 15 control samples (approx. 2%). 1,211 aberrations >50 kb were retained. In a gene-centric approach we also excluded aberrations which only affected intronic or intergenic sequences. The remaining 733 CNVs were reviewed for gene content and familial segregation with the growth phenotype either by array analysis or by multiplex ligation-dependent probe amplification (MLPA; Table S1). We retained all CNVs that were either *de novo* in the sporadic cases or co-segregated with the phenotype in the familial cases. In addition both groups had to meet at least one of the following criteria: a) CNVs with previously described human growth phenotypes of the affected genes obtained from the OMIM database, b) Murine knock-out phenotypes of the Mouse Genome Informatics database (http://www.informatics.jax.org) including keywords like growth retardation and decreased body size (Table S2), c) genes with a possible role in height development and/or bone growth based on their reported function on cell cycle regulation, organization of the cytoskeleton, chromatin remodeling, cilia development and the involvement in important developmental pathways (Table S3), d) loci overlapping non-polymorphic, gene-containing aberrations of the Decipher database (http://decipher.sanger.ac.uk) with short stature as one of the described phenotypes.

Taken together all lines of evidence we identified a total of 20 likely pathogenic copy number changes, 10 deletions and 10 duplications, in 20 families (10% of the study group) (Table 2). It is striking that in the RefSeq exons covered by all 20 CNVs we found no overlapping control CNVs at all in 19 and just one control CNV overlapping some exons in the 5p15.33 CNV (Table S3). The size of these 20 CNVs ranged from 109 kb to 14 Mb. All 20 CNVs were independently confirmed by MLPA (Figure S2A–S2T). 7 aberrations (35%), 4 deletions and 3 duplications, were *de novo* (parental relationships confirmed) with an average size of 2,594 kb and an average of 30 genes. As we expected 6×10^−3^ de novo CNVs per haploid genome per generation in the healthy population [20], the identified number of de novo CNVs>50 kb in our patients was significantly higher than expected by chance further supporting pathogenicity of these variants (p-value 0.03, Fisher's exact test).

**Table 2:** Lines of Evidence.

Patient	Inheritance	Gain/Loss	Locus	Position hg19 (Mb)	Size (kb)	# of affected genes	SOS genes^1^	MD syndromes^2^	# of genes with growth function^3^	GEPIS-Tissue^4^	Decipher^5^	Significant differentially expressed genes (all genes)	Mouse genome Database^6^	GWAS^7^	Breakpoint disrupting gene	HI score^8^
1	de novo	loss	1q32.1	chr1:204.2–206.6	2157	33	-	-	5	6	0	10 (33)	3	4.5×10^−4^	PLEKHA6, SRGAP2	3
2	de novo	loss	2q36.1–36.3	chr2:221.9–228.6	6695	31	PAX3	-	3	8	2	7 (28)	4	2.17×10^−8^*	-	5
3	de novo	loss	14q23.1	chr14:57.1–58.4	1352	8	-	-	1	3	0	2 (8)	1	1.19×10^−2^	C14orf37	3
4	de novo	loss	22q11.21–11.22	chr22:21.6–22.9	1363	23	-	22q11.2	5	5	4	n/a	2	1.1×10^−3^	-	3
5	de novo	gain	2p23.3	chr2:25.2–29.7	4573	77	POMC, GCKR	-	13	21	0	24 (77)	7	2.79×10^−13^*	-	8
6	de novo	gain	19q13.43	chr19:58.3–58.7	367	13	-	-	10	3	0	1 (13)	1	1.26×10^−1^	-	0
7	de novo	gain	3q29	chr3:195.7–197.3	1653	29	-	3q29	5	4	0	11 (29)	5	8.33×10^−3^	-	3
8	maternal	loss	1q21.1	chr1:146.1–148.6	2116	26	-	1q21	3	2	3	n/a	0	1.42×10^−6^	-	2
9	maternal	loss	22q11.22	chr22:22.3–22.5	259	1	-	22q11.2	1	0	2	0 (1)	0	4.29×10^−2^	TOP3B	0
10	maternal	gain	17q11.2	chr17:30.8–31.2	393	2	-	-	0	2	0	n/a	0	5.57×10^−2^	-	0
11	maternal	gain	5q22.1-q23.2	chr5:110.0–124.2	14229	59	-	-	12	17	0	n/a	7	2.08×10^−8^*	-	2
12	maternal	gain	1q21.1	chr1:145.6–145.9	307	6	-	1q21.1/TAR	0	1	1	3 (6)	0	2.39×10^−3^	-	0
13	maternal	gain	2q33.2	chr2:203.4–203.7	323	3	-	2q33	1	1	0	n/a	1	1.01×10^−4^	-	1
14	maternal	gain	7q36.3	chr7:156.9–157.1	165	1	-	-	0	1	1	n/a	0	1.13×10^−2^	-	0
15	maternal	gain	1p36.33	chr1:0.9–1.2	306	20	-	1p36	1	3	0	n/a	1	5.25×10^−2^	-	1
16	maternal	gain	2q21.2	chr2:133.1–133.7	598	3	-	-	0	0	1	n/a	0	8.76×10^−4^	-	0
17	paternal	loss	13q22.1	chr13:73.4–73.5	118	1	-	-	0	0	2	0 (1)	0	1.43×10^−2^	PIBF1	0
18	paternal	loss	14q21.1-q21.2	chr14:40.2–42.1	1871	1	-	-	0	0	1	0 (1)	0	4.32×10^−2^	LRFN5	0
19	paternal	loss	1q21.1	chr1:145.9–147.8	1654	17	-	1q21	3	2	3	2 (15)	0	1.42×10^−6^	-	2
20	paternal	loss	5p15.33	chr5:0.6–0.7	109	1	-	-	1	0	0	n/a	0	1.11×10^−1^	TPPP	0

1 Known short stature causing genes.

2 MD = Microdeletion/Microduplication syndromes;

3 Details in Table S3;

4 # of genes expressed in relevant growth tissues (bone, cartilage) according to GEPIS-tissue;

5 Patients in Decipher with overlapping CNVs and short stature;

6 # of genes with short stature in the mouse model,

7 min p-value in GIANT association study;

8 # of genes with Haploinsufficiency score<10%.

Unlike other entities with reduced reproductive fitness e.g. severe intellectual disability with a high rate of *de novo* CNVs [21], [22], we anticipated a higher rate of inherited CNVs in short stature as no reproductive disadvantage is known. This was confirmed by the identification of 13 inherited CNVs with an average CNV size of 1,727 kb and an average of 10 genes.

**Figure 4:**
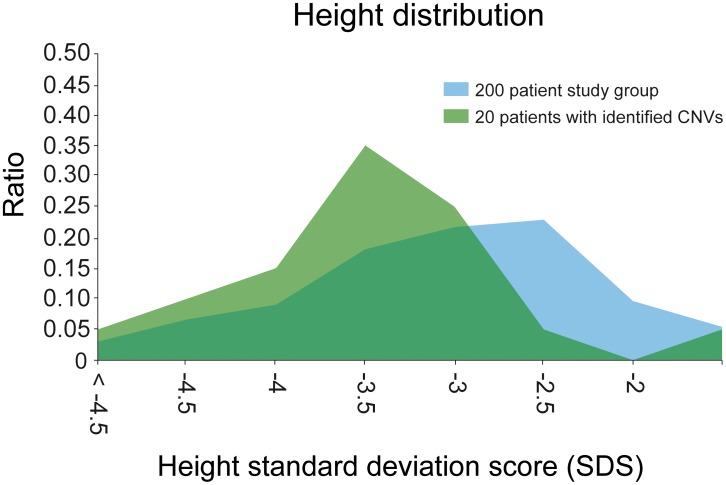
Height distribution of the study group. Height distribution (SDS) of all 200 patients (blue) compared to the 20 patients with identified CNVs (green). The mean SD score for height was −3.34. Patients with identified CNVs showed a significant difference in the SDS distribution (p-value 0.03).

This group of 20 affected individuals with highly probable pathogenic CNVs consisted of 9 male and 11 female individuals (Table 3). Interestingly, the mean SD score for height was −3.34 and the SD score distribution of these 20 individuals was significantly lower when compared to the total study group (p-value 0.009; Wilcoxon test) (Figure 4). Thus, rare pathogenic CNVs are more likely identified in patients with severe short stature. No significant difference was observed in patients with CNVs with prenatal vs. postnatal onset, proportionate vs. disproportionate growth retardation, and syndromic vs. non-syndromic short stature (Table S4), but the number of affected cases in each group was small with limited statistical power. However, 40% of the 20 patients had a prenatal onset of short stature compared to 26% of the entire study group indicating central regulatory pathways of embryonic development to be disturbed by genes located in these CNVs.

**Table 3:** Summary phenotype of patients with identified CNVs.

		Segregation	
Feature	total	de novo	inherited
Number of patients	20	7	13
Male/Female	9/11	4/3	5/8
Mean standard deviation score (SDS)	−3.34	−3.84	−3.07
Proportionate/disproportionate	16/4	7/0	9/4
Syndromic/isolated	8/12	3/4	4/9
Intellectual status normal/mild learning disability	11/9	3/4	8/5
Primordial growth retardation yes/no	8/12	2/5	6/7
Mean aberration size (kb)	2030	2594	1727

Eight patients had CNVs showing an overlap with 6 known microdeletion/duplication syndromes associated with short stature (Table 2). Two of these patients (patient 8 and 19) had large inherited deletions covering the complete 1q21.1 microdeletion region which is known for its phenotypic variability [23]. Short stature is present in about 25–50% of the patients [24]. Further commonly observed signs such as mild facial anomalies, microcephaly and developmental delay were also observed in our patients. The inherited 307 kb duplication of patient 12 included the distal end of the TAR syndrome susceptibility locus on 1q21.1 but without the recently reported *RBM8A* gene region [25]. A 1.6 Mb duplication overlapping the rare 3q29 microdeletion/duplication syndrome was found *de novo* in one patient with non syndromic idiopathic short stature (patient 7). Features of the 3q29 duplication syndrome have not been clearly determined, but failure to thrive has occasionally been reported [26]. We also found a 1,363 kb *de novo* deletion partially overlapping the classical and distal 22q11.22 microdeletion region of DiGeorge/Velo-cardio-facial syndrome [27]–[29] and a 259 kb inherited deletion within the distal part of 22q11.22 only (patient 4 and 9, respectively) [27]. Correspondingly, these two patients presented with short stature and some mild facial features, but no cardiac defects. Inherited duplications in patient 13 and 15 slightly overlapped the microdeletion regions 2q33 and 1p36 [30], [31]. Thus, the clinical presentation of these patients confirmed the broad variability of known microdeletion/duplication syndromes and might highlight potential candidate genes for the short stature phenotype in these entities.

**Figure 5:**
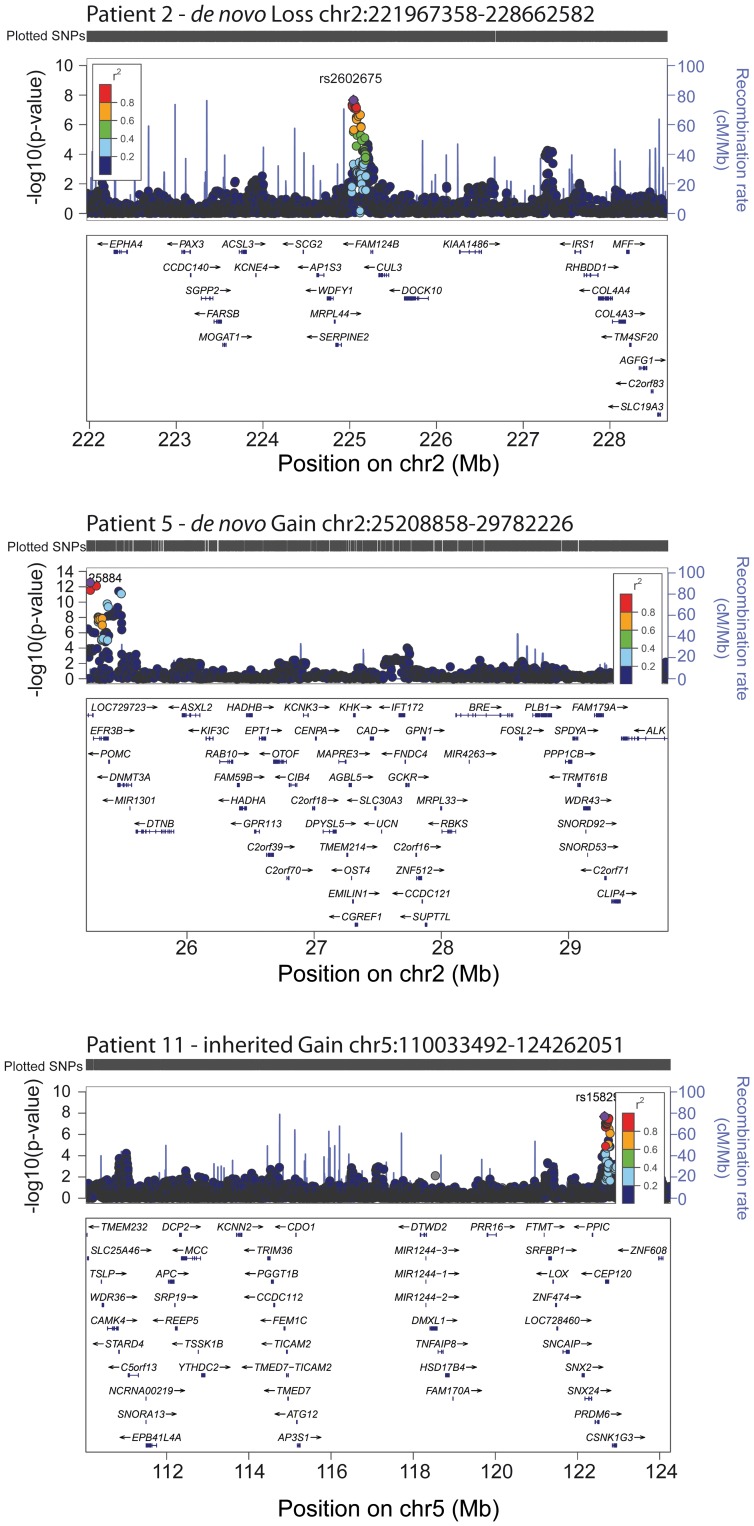
Genome-wide significant association of GWAS loci for height distribution in the 3 CNVs. The values of r^2^ are based on the CEU 1000genomes Nov 2010 samples. The blue line and right-hand y axis represent recombination rates. The SNPs with the min p values are highlighted as purple diamond. The figures were created using LocusZoom (http://csg.sph.umich.edu/locuszoom/).

Recent genome-wide association studies (GWAS) found common single nucleotide polymorphisms (SNPs) in at least 180 loci to be significantly associated with height variation in the general population. These associated loci accounted only for up to 10% of the phenotypic variation within the normal range of the Gaussian growth distribution [32]. We investigated if these loci might be located within our identified rare CNVs. Using LocusZoom [33] we compared position and gene content with the published genome wide association dataset of the GIANT consortium based on the CEU 1000genomes Nov 2010 imputation. To identify significant loci, we considered a Bonferroni corrected level of significance of 1.377×10^−6^ based on 36,316 SNPs from the GIANT dataset located in the 20 identified CNVs. Loci at 3 of our CNV regions reached this level of significance (variants with the best p-values respectively r^2^ values are shown in Figure S3A–S3T and Table S5). The loci with the best p-values were located in the 6.7 Mb deletion 2q36.1–36.3 (patient 2) (Figure 5), 4.5 Mb duplication 2p23.3 (patient 5), and 14.2 Mb duplication 5q22.1-q23.2 (patient 11). rs11125884 located in the promoter region of *EFR2B* (patient 5) even reached a level of genome wide significance based on SNP association (2.8×10^−13^). This number of 3 CNVs with significant associated SNP loci out of the 20 likely pathogenic CNVs was significantly higher than expected by chance (p-value<1×10^−3^). Our findings not only confirmed the significance of the published results of the genome wide association study but also suggest a possible functional link between common variants in growth variation and rare variants involved in severe growth retardation underlining a major gene effect in short stature.

A deletion or duplication of one gene or a subset of genes located in a CNV can lead to directly or indirectly impaired gene expression [34], [35]. To investigate whether this is the case for the 20 identified CNVs we performed expression profiling in 11 individuals where RNA from lymphocytes was available. Of the 188 genes contained in the CNVs 58 (31%) showed a significant differential gene expression in the direction of the respective CNV (Table S6). This number of differentially expressed genes we observed would be expected by chance only with a probability of less than 0.001 according to the binomial distribution, suggesting that these genes are dosage sensitive and the identified aberrations are leading to haploinsufficiency of these genes. To explore whether these 58 differentially expressed genes cluster in networks known to be involved in growth we performed pathway analyses using Ingenuity Pathway Analysis (IPA). This analysis identified networks involving cell death, cell cycle and DNA repair (Table S7) further supporting the pathogenicity of these CNVs.

In conclusion, we propose rare CNVs as a relatively common cause of short stature under a major gene effect model. These include duplications as well as deletions of more than 100 kb in a comparable frequency as observed in other entities e.g. intellectual disability [12], [36]. Our findings also provide strong evidence for a “rare variant – frequent disease” hypothesis for short stature.

## Materials and Methods

### Patient cohort

All individuals gave their consent to this study, which was approved by the Ethical Review Board of the Friedrich-Alexander University Erlangen-Nuremberg. Phenotypic data, medical history, and family history of 200 affected individuals with short stature and their family members were ascertained and compiled in a database. In all cases photographic documentation, pediatric and partially radiological evaluation was available. Height adjusted SD scores were calculated on the basis of the Prader Growth charts. A control cohort used to exclude common copy number polymorphisms consisted of 820 individuals originating from the same Central European region with either exfoliation syndrome or psoriatic arthritis. DNA and RNA of the affected individuals and DNA of their respective parents were obtained. DNA of cases and controls was extracted from blood samples using the same method and all necessary quality assessments during DNA sample and array preparation were attributed. Principal component analysis of the 200 affected and 820 control individuals demonstrated minor ethnic heterogeneity within both cases and controls caused by a residual amount of ethnic heterogeneity in both populations (Figure S4). This was comparable in both and considered not relevant for the identification of rare variants in contrast to the classical test of association not applied in this study.

### Molecular karyotyping

Molecular karyotyping was performed using Genome-Wide Human SNP 6.0 and CytoScan HD arrays (Affymetrix, Santa Clara, USA). We calculated the genome wide copy number using the Genotyping Console 3.0.2 and the Chromosome Analysis Suite v1.2.2 software (Affymetrix, Santa Clara, USA). To exclude batch effects, all cases and controls were processed randomly. In-house quality criteria of a contrast QC value >0.4 and a MAPD value <0.4 were met by all 200 samples. Instead of the Affymetrix reference model file, our own reference file was created using 167 healthy control samples on the same platform with equal conditions. Copy number variants with a minimum of 10 kb and 5 affected markers (Genome-Wide Human SNP 6.0) or 20 affected markers (CytoScan HD array) were calculated. As CNV calling for the Y chromosome was not reliable, CNVs on the Y chromosome were excluded from further investigation.

At first a screening of all identified CNVs above 50 kb against an independent control cohort of 820 healthy control individuals excluded common copy number polymorphisms. 50 kb was the observed minimum resolution of the arrays where independent validation leads to reliable results. Accordingly, aberrations which showed a size overlap of more than 95% in more than 15 control samples were automatically excluded from further investigation. CNVs with less than 95% overlap in control samples but where all included genes are covered in CNVs of at least 15 control samples were also removed. As we aimed for rare variants all remaining CNVs were compared with CNVs annotated in the Database of Genomic Variants to exclude common variants which might not be covered by CNVs of our 820 control individuals. No additional CNVs were excluded in this step.

### CNV validation

Multiplex ligation-dependent probe amplifications (MLPAs) were carried out using self-made MLPA kits with the SALSA MLPA Reagents kit and the P200 SALSA MLPA Reference kit according to the manufacturer's instructions (MRC Holland, Amsterdam, Netherlands). The reference kit includes control fragments and reference probes from 172 to 250 nucleotides. Corresponding to the affected copy number regions, MLPA probes were designed as described in the general guidelines of synthetic probe design by MRC Holland with the hg18 version of the human reference sequence (NCBI36, March 2006). For all oligonucleotides the RAW program was used to ensure a melting temperature of ≥70°C and a GC content of about 40–60%. The absence of annotated SNPs and a unique hybridization site was verified with the BLAT program. Including universal primer binding sites, the synthetic oligonucleotides product size was within a range of 100–136 bp. For the purpose of ligation all right probe oligos (RPOs) were 5′ phosphorylated (Thermo Fisher Scientific, Waltham, USA). 250 ng of genomic DNA were used to carry out the MLPA reaction. The PCR products were separated according to their length by capillary electrophoresis on an ABI PRISM 3100 Genetic Analyzer. The corresponding copy number of each locus was calculated by comparing the relative peak area of every patient to the mean peak area of at least 5 control individuals (probe ratios) in the MLPA module of the Sequence Pilot software (JSI medical systems GmbH, Kippenheim, Germany). Probe ratios under 75% indicated a deletion, whereas ratios over 125% confirmed duplications.

Furthermore, segregation of copy number changes in the families was confirmed by MLPA testing of the parental DNAs. CNVs inherited from unaffected parents were excluded from further investigation.

### Association analysis

For the genome-wide copy number association analysis, pseudomarkers were defined at the endpoints of each CNV segment and the copy number status at this marker determined for each individual. Gains and Losses were separately evaluated in a permuted χ^2^ based test with 100,000 status permutations performed for each marker. Association analysis was performed with PLINK v. 1.07 [37] and visualized with GPGraphics [38].

### Expression arrays

The PAXgene Blood RNA Kit by Qiagen was used to extract RNA from peripheral blood samples of the affected individuals. Available RNAs of 14 of the 20 affected individuals with proposed pathogenic CNVs were controlled for quantity and quality with an Agilent 2100 Bioanalyzer (Agilent, Santa Clara, USA). When quality standards were not passed, an additional purification was carried out using the “RNeasy Mini Kit” according to the manufacturer's instructions (Qiagen, Hilden, Germany). RNA of 3 individuals did not achieve the necessary quality requirements and were excluded from further analysis. Gene expression arrays of the remaining 11 affected individuals and 5 healthy controls were performed using the Affymetrix GeneChip Human Genome U133 Plus 2.0 Array. Expression data was analyzed with the PARTEK software (Partek Incorporated, St. Louis, USA). After GCRMA normalization, a one-way ANOVA analysis was performed to test for significant differences in gene expression levels.

### Pathway analysis

Ingenuity Pathway Analysis (IPA) (Ingenuity Systems Inc., Redwood City, USA) was used to analyze potential functional relationships between affected genes of all 20 identified CNVs. A list of all affected genes (278 unique) as well as lists of all (60 unique), only deleted (21 unique), and only duplicated genes (39 unique) showing significant (p<0.05) differential expression of the 11 patients with expression array data available were analyzed. The fold change minimum was set to 1.25 (up regulation) and −1.33 (down regulation), respectively. After uploading the individual lists the software clustered genes according to their connectivity into molecular networks, common biological functions and canonical pathways. P-values and numerical scores were calculated to rank networks according to their degree of relevance in regards to the different gene lists. The calculated score is based on the hypergeometric distribution with the right-tailed Fisher's Exact Test. The score presents the negative log of the p-value.

### GWAS comparison

We compared the position and gene content with the published genome wide association data of the GAINT consortium (CEU 1000genomes Nov 2010 sample imputation) using LocusZoom [32], [33]. Under the estimation of approx. 2000 copy number polymorphism of 50 kb or above in the human genome per individual we calculated a genome wide level of significance of 2.5×10^−5^.

## Supporting Information

Figure S1 Copy number association of 200 patients and 820 control individuals. One-sided test of association of deviant copy number state at a given location and disease status, both for deletions (left) and duplications (right) after calculating the negative decadic logarithms of corrected (Bonferroni for 17,168 tests) permutation-based χ^2^ p-values (10,000,000 permutations). The horizontal line represents the estimated significance threshold of genome wide CNV association (p-value 2.5×10^−5^). One significant deletion locus on chromosome 1 did not harbor genes or regulatory elements with supportive evidence for an effect on height.
(DOCX)

Figure S2 Graphical presentation of the copy number state and MLPA confirmation. (A-T, upper pane) Presentation of the calculated copy number for the patient (green), the mother (magenta) and the father (blue) where available (Affymetrix Genotyping console). (A-T, lower pane) MLPA confirmation of one gene in each of the affected CNV regions vs. controls (C1-12). MLPAs were carried out using the SALSA MLPA Reagents kit and the P200 SALSA MLPA Reference kit according to the manufacturer's instructions (MRC Holland, Amsterdam, Netherlands). At least one MLPA probe per CNV was designed. The corresponding copy number of each locus was calculated by comparing the relative peak area of every patient to the mean peak area of the control individuals (probe ratios) with the Sequence Pilot software (JSI medical systems GmbH, Kippenheim, Germany).
(DOCX)

Figure S3 GWAS loci in the identified 20 potential causal CNVs. (A–T) The r^2^ values in the figure refer to the LD between the GIANT SNPs based on the CEU 1000genomes Nov 2010 samples. The blue line and right-hand y axis represent recombination rates. The SNP with the best p-value representing the best r^2^ value is highlighted as purple diamond. The figures were created using LocusZoom (http://csg.sph.umich.edu/locuszoom/).
(DOCX)

Figure S4 Principal component analysis of 200 patients (red and green) and 820 control individuals (blue). In the lower panel, the area containing most individuals in the upper panel has been expanded. In both groups, some samples scatter along the first principal component, owing to a residual amount of ethnic heterogeneity in both populations. Green data points indicate the patients with potentially disease-causing copy number variants.
(DOCX)

Table S1 MLPA probes.
(DOCX)

Table S2 Murine knock-out phenotypes of the Mouse Genome Informatics database (MGI) and Haploinsufficiency scores of genes within the identified CNVs.
(DOCX)

Table S3 Candidate genes based on their function.
(DOCX)

Table S4 Detailed clinical data of patients with the identified 20 CNVs.
(DOCX)

Table S5 Association results for the identified CNVs from genome wide association of human height variation GIANT consortium data. Signals with p values<1.377×10^−6^ are marked (*).
(DOCX)

Table S6 Significant differentially expressed candidate genes within the identified CNVs.
(DOCX)

Table S7 Network analysis of affected genes within the identified candidate CNVs.
(DOCX)
