# Supplementary figures and images for: Rare Copy Number Variants Are a Common Cause of Short Stature
Source: PLoS Genet. 2013 Mar 14;9(3):e1003365. doi: 10.1371/journal.pgen.1003365 (PMC3597495; doi:10.1371/journal.pgen.1003365)

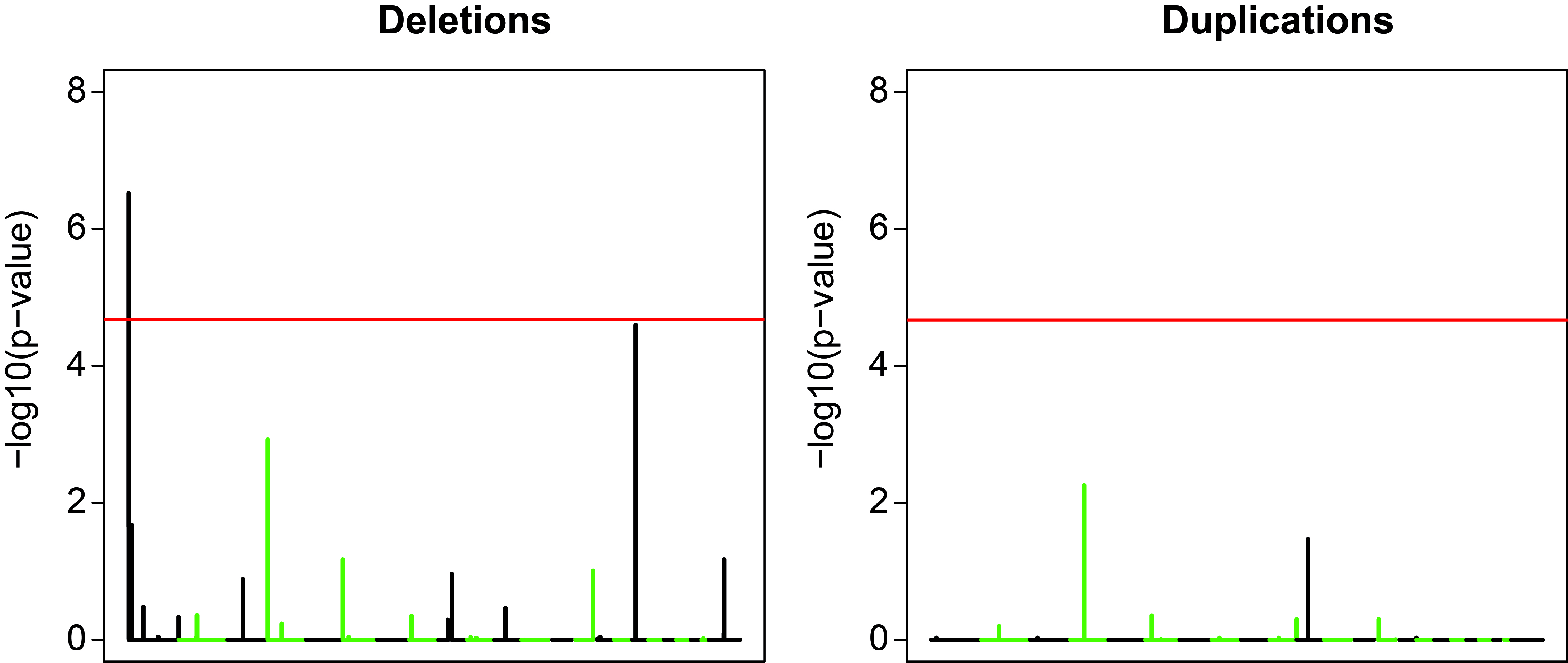

Supplement: Figure S1 — Copy number association of 200 patients and 820 control individuals. One-sided test of association of deviant copy number state at a given location and disease status, both for deletions (left) and duplications (right) after calculating the negative decadic logarithms of corrected (Bonferroni for 17,168 tests) permutation-based χ2 p-values (10,000,000 permutations). The horizontal line represents the estimated significance threshold of genome wide CNV association (p-value 2.5×10^−5^). One significant deletion locus on chromosome 1 did not harbor genes or regulatory elements with supportive evidence for an effect on height. (DOCX) [file pgen.1003365.s001.docx]

A


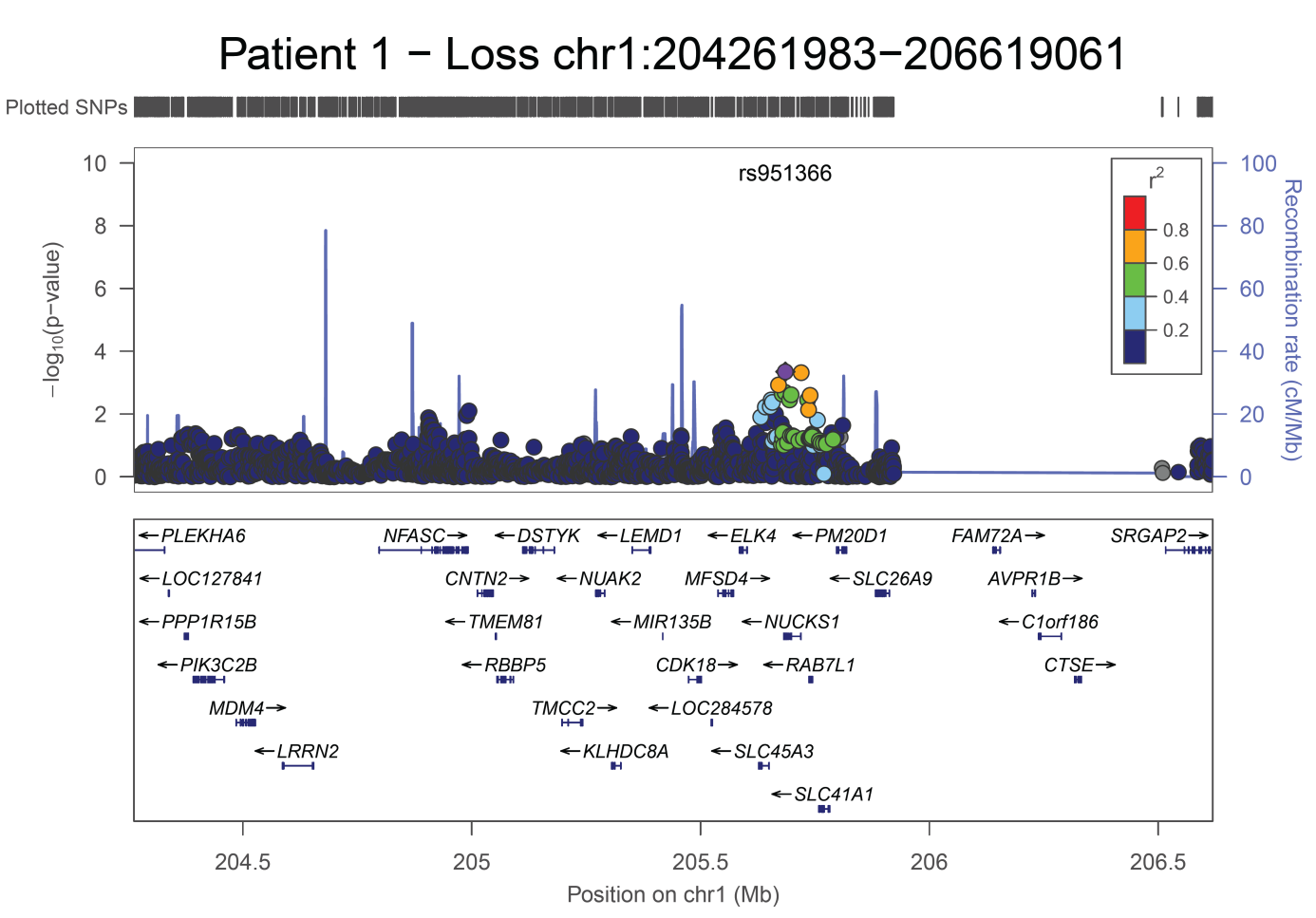


B


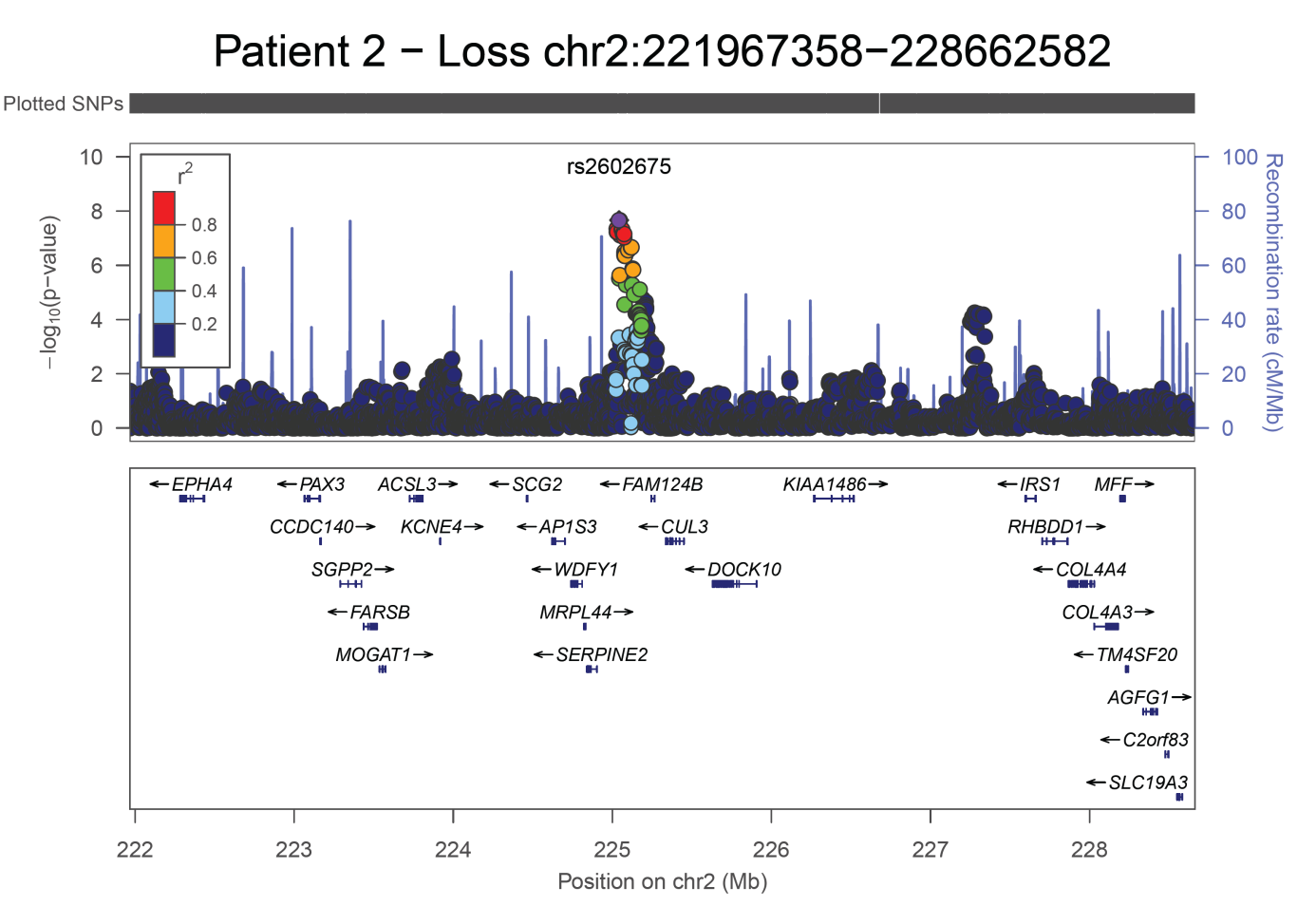


C


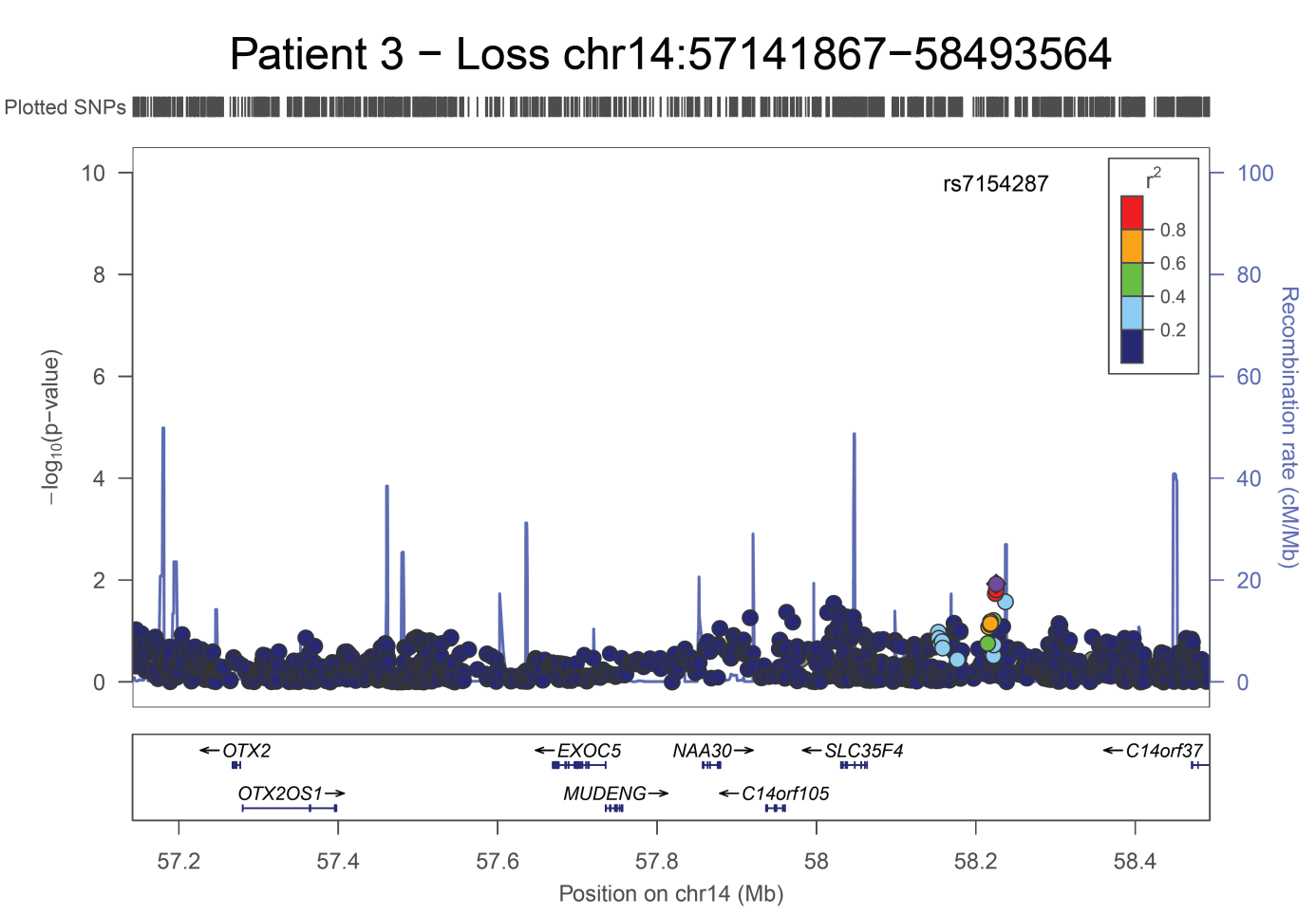


D


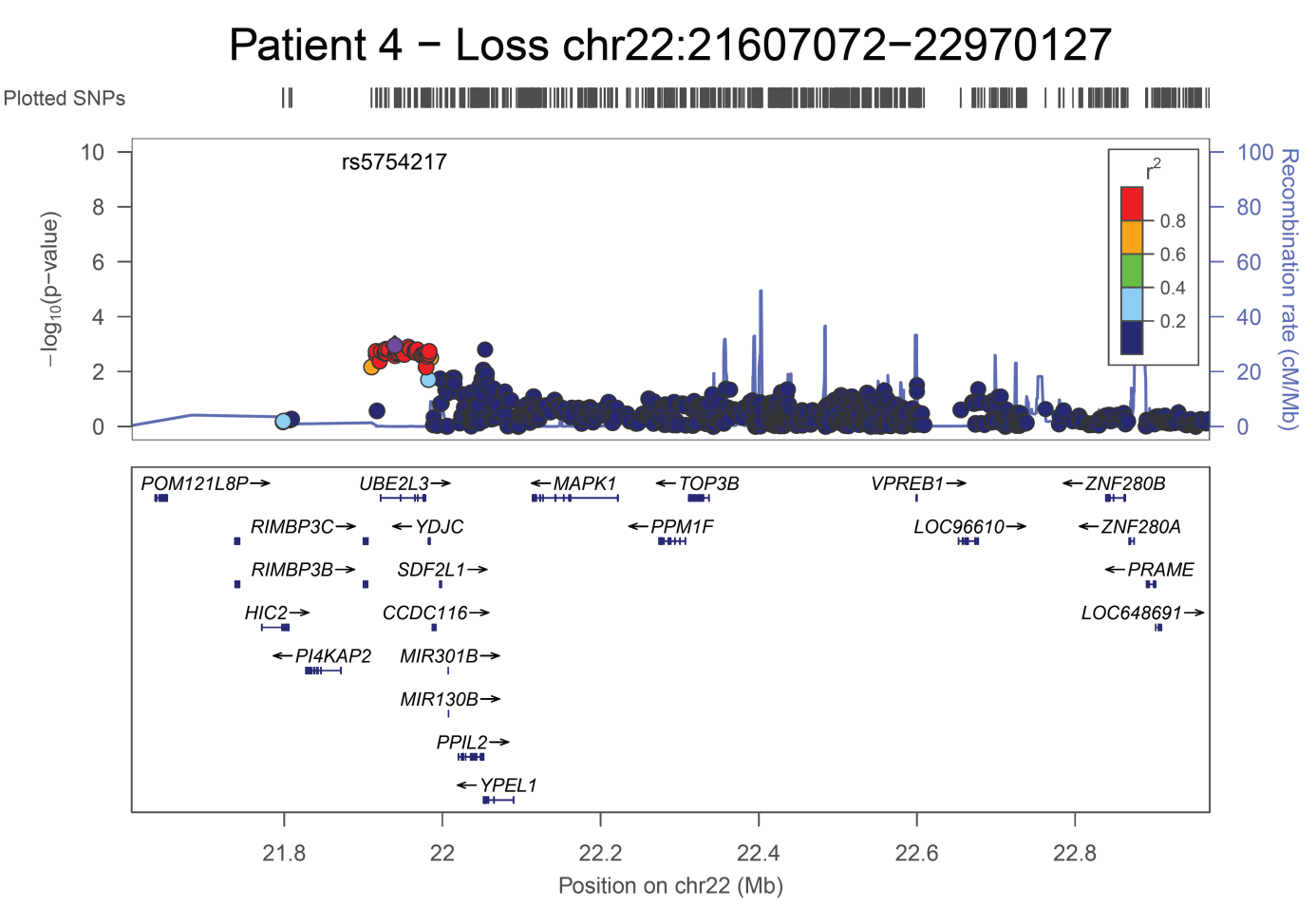


E


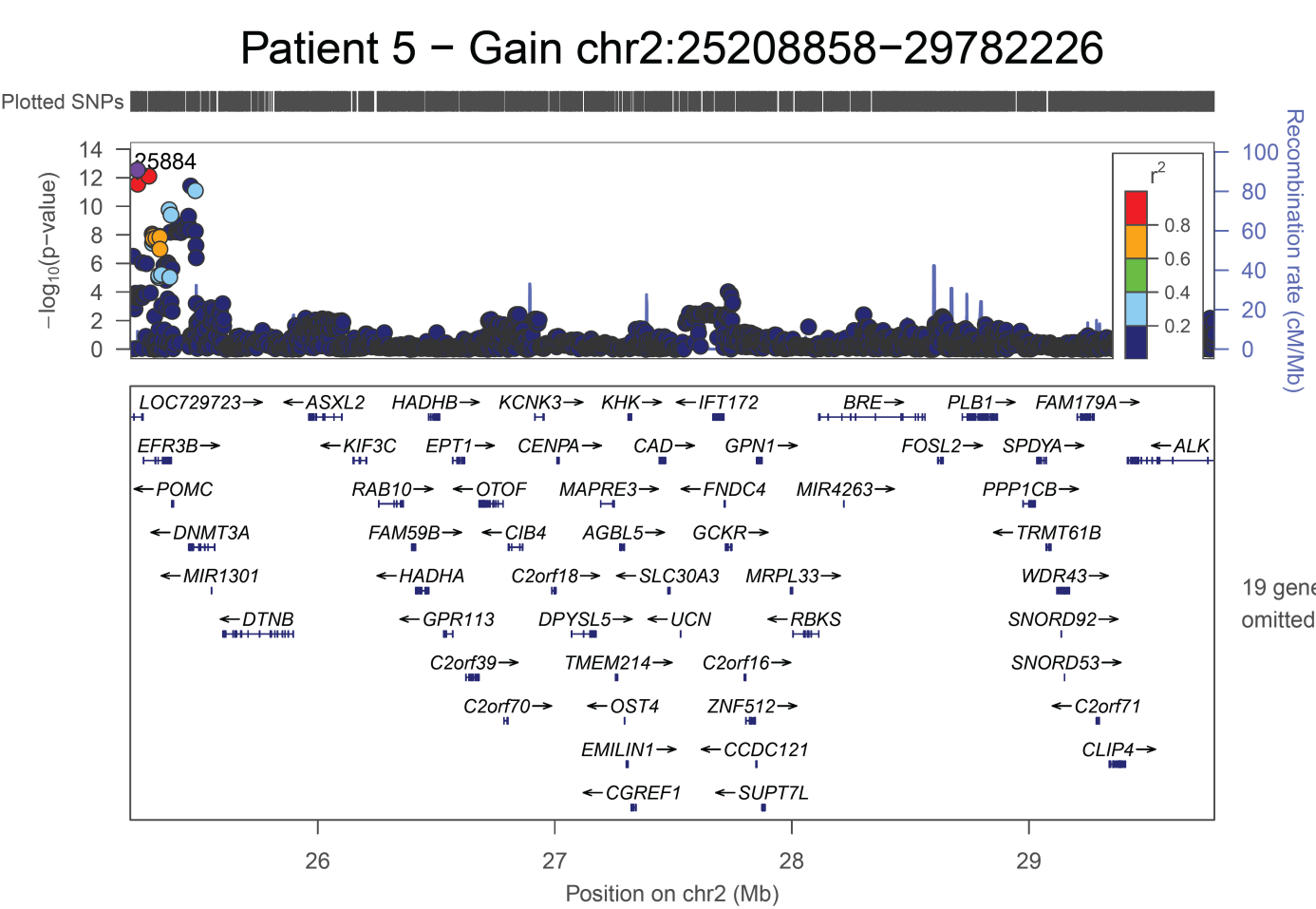


F


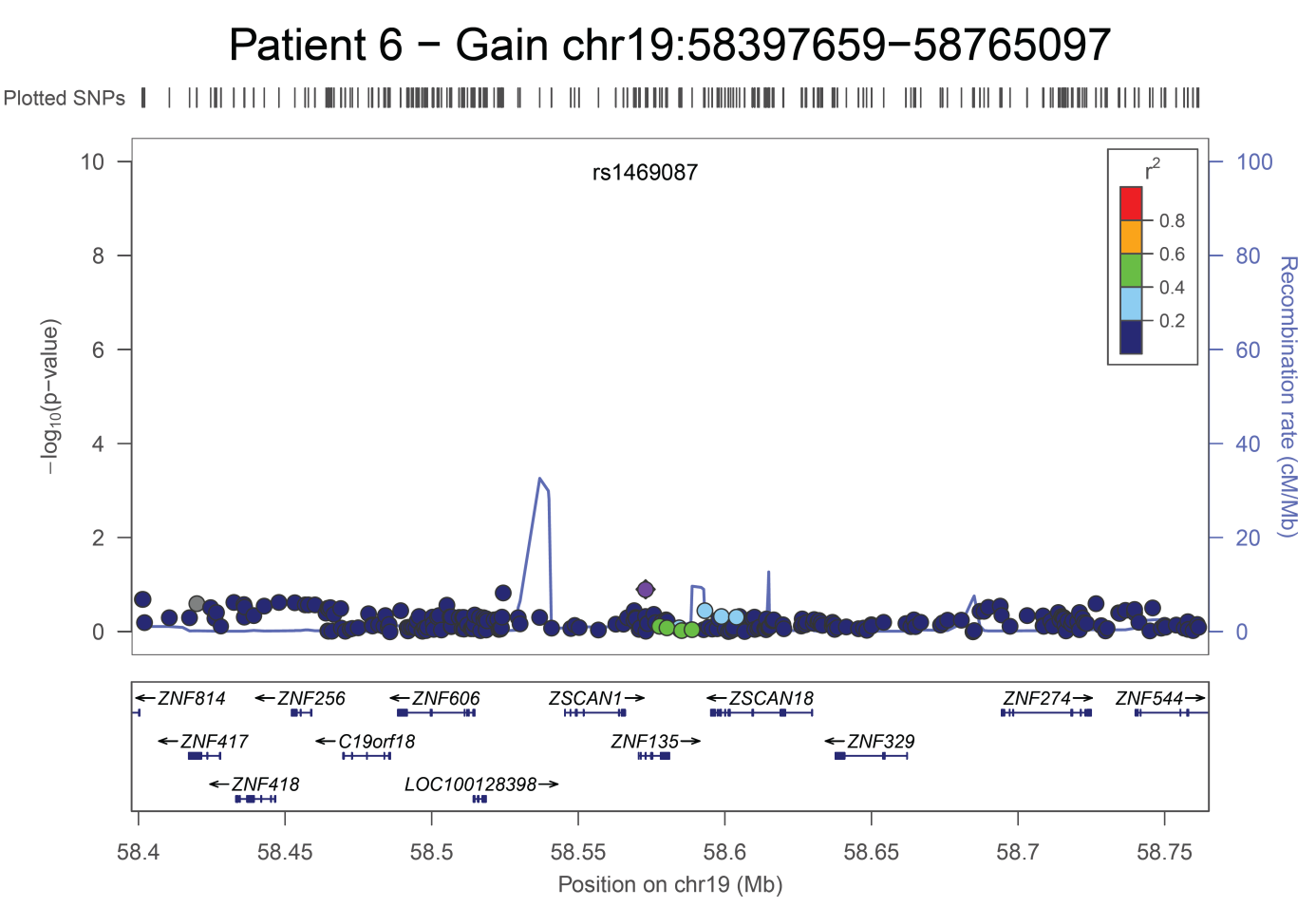


G


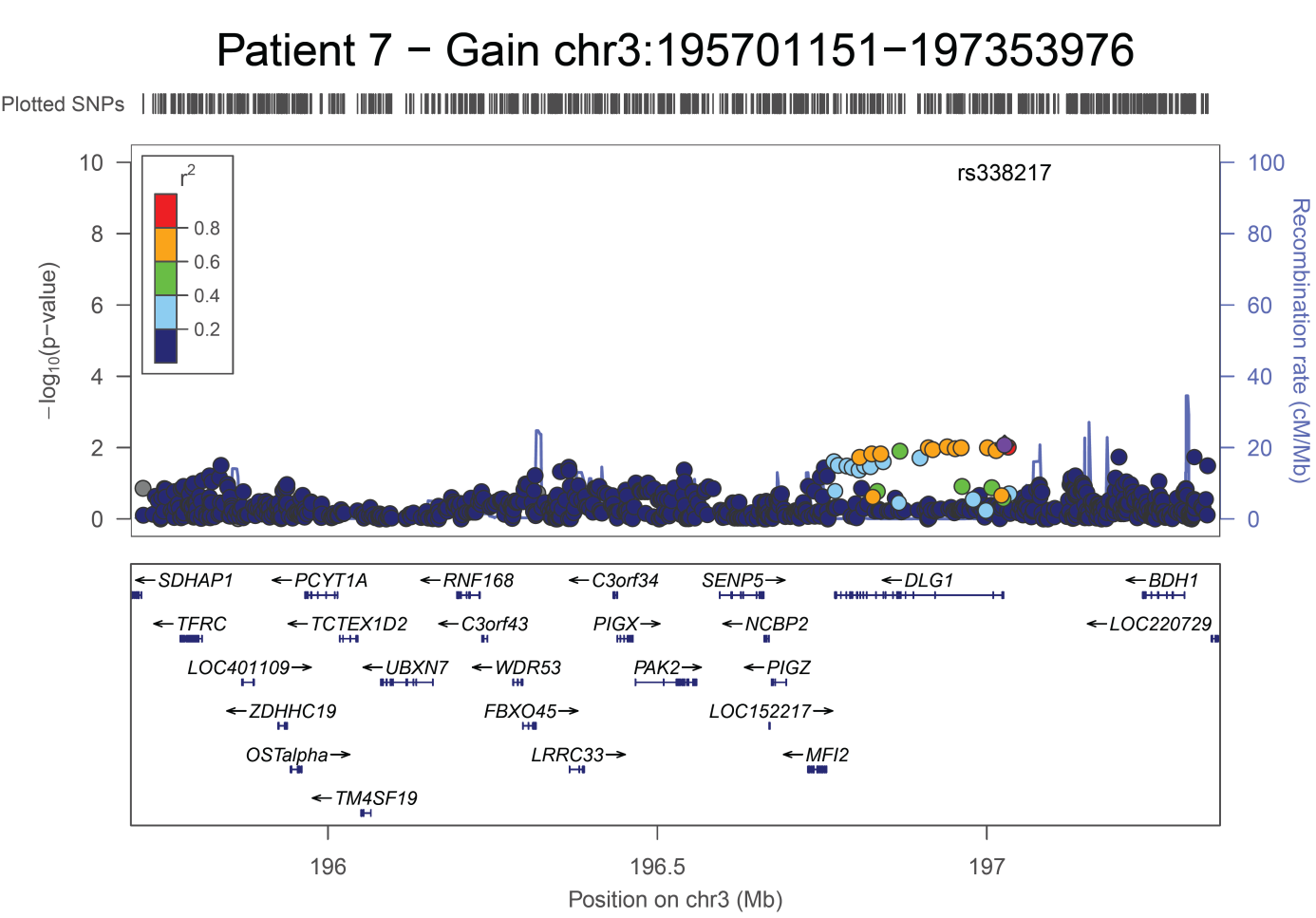


H


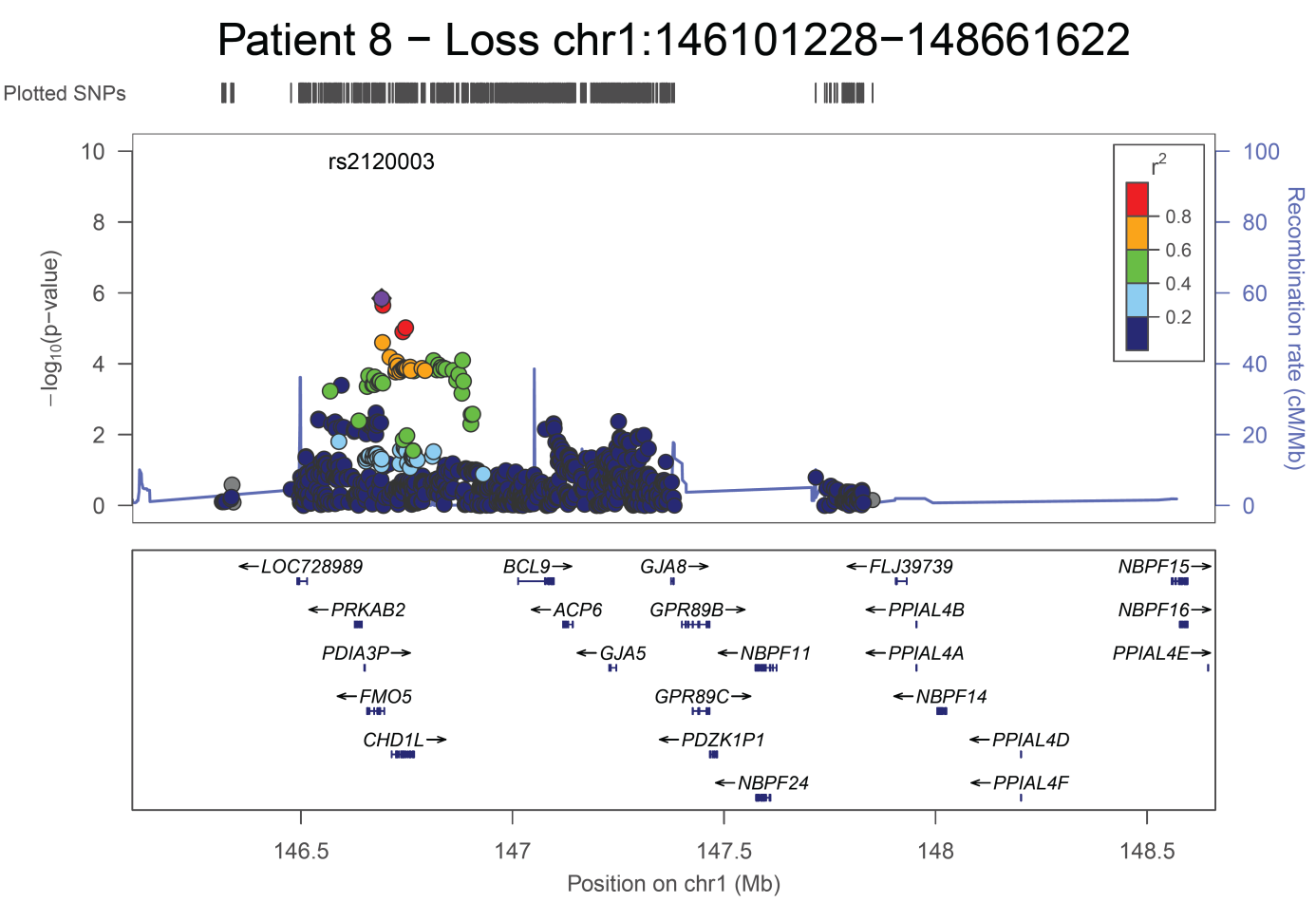


I


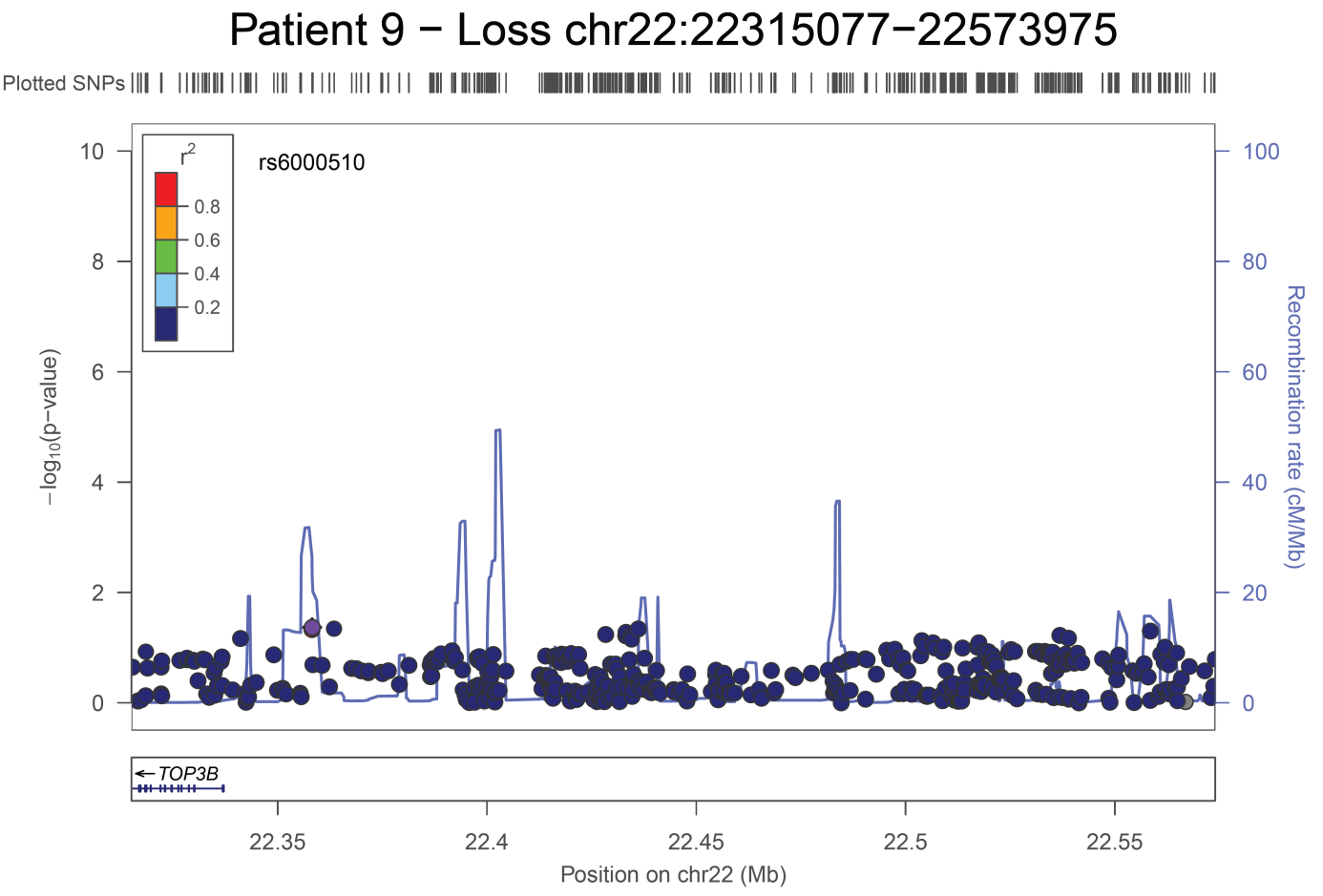


J


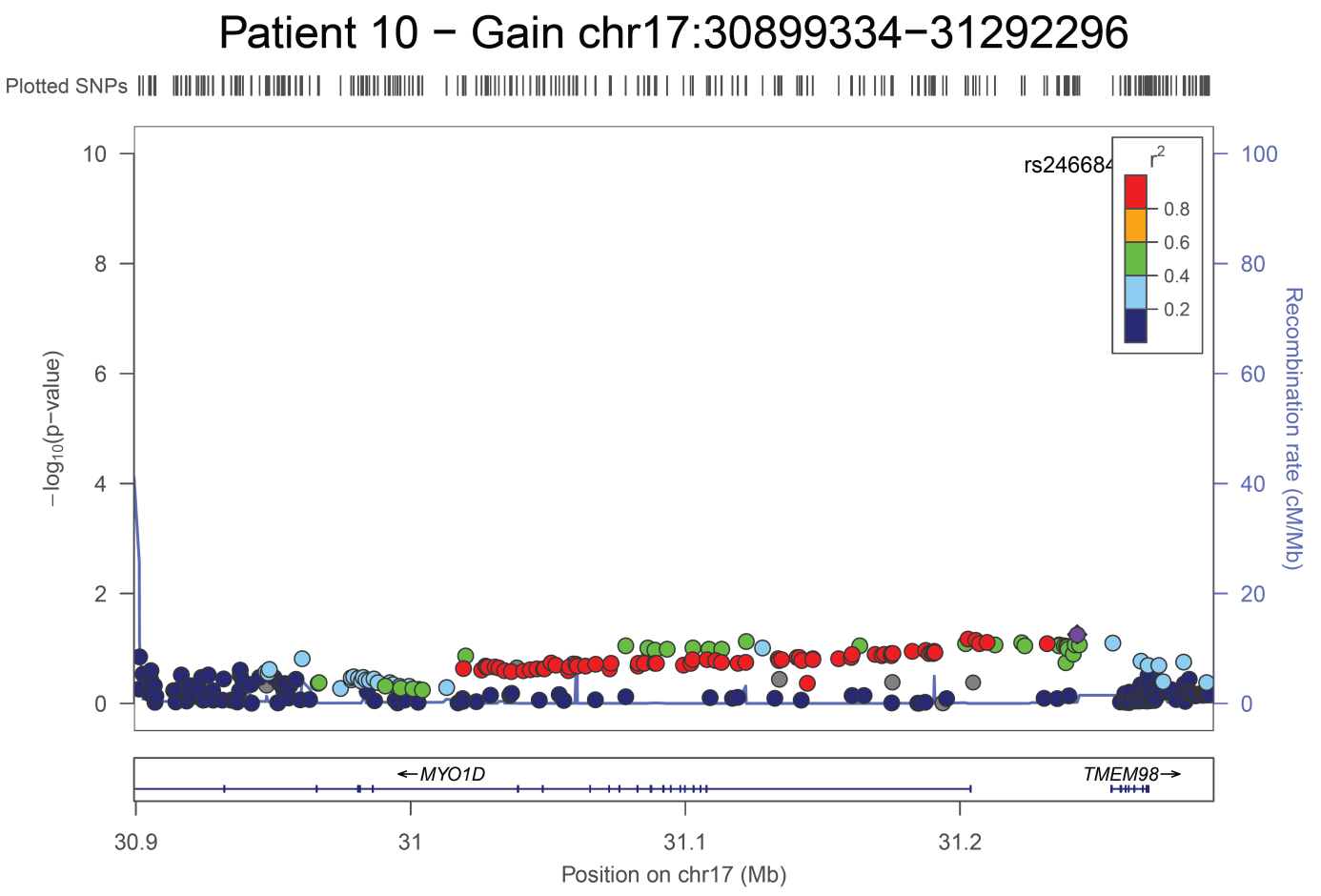


K


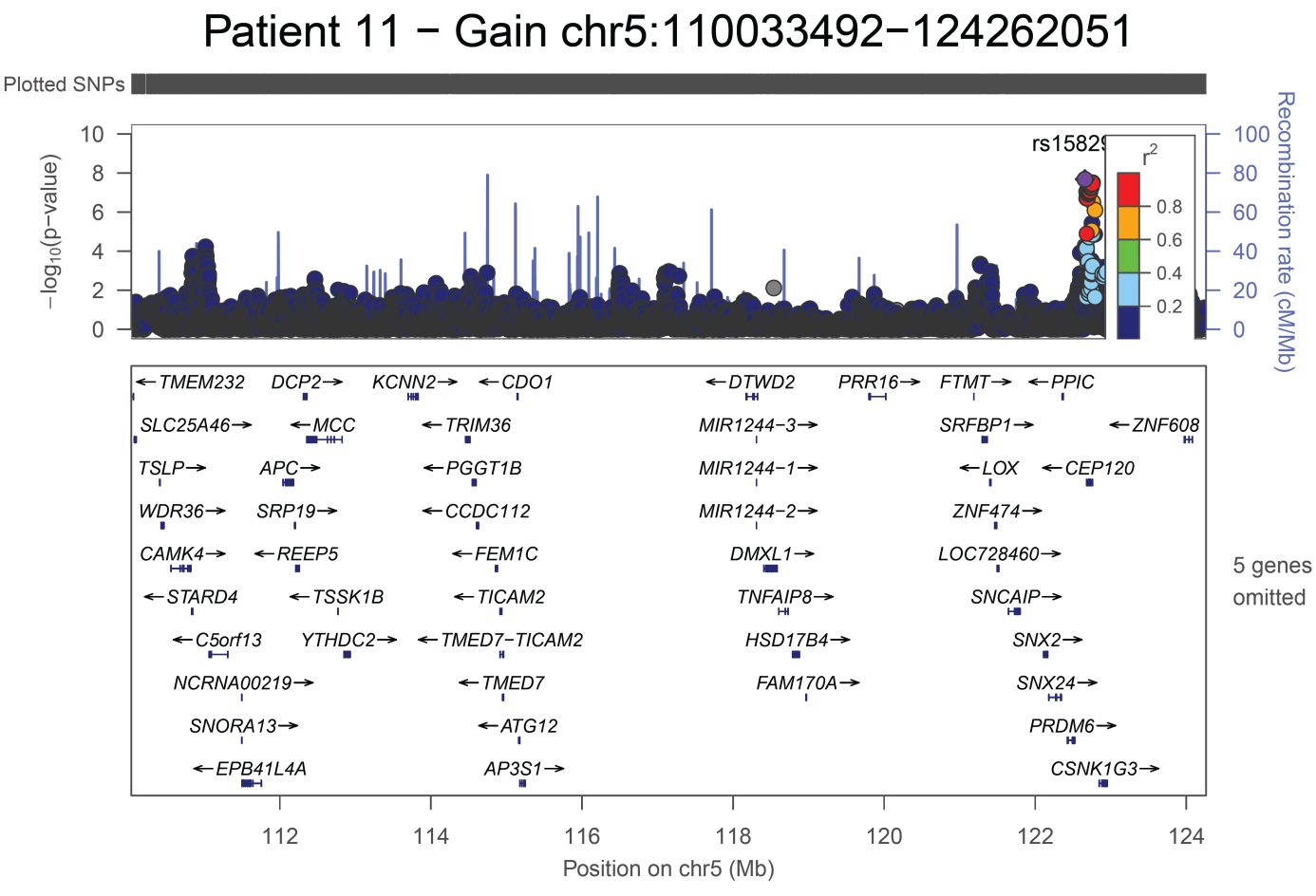


L


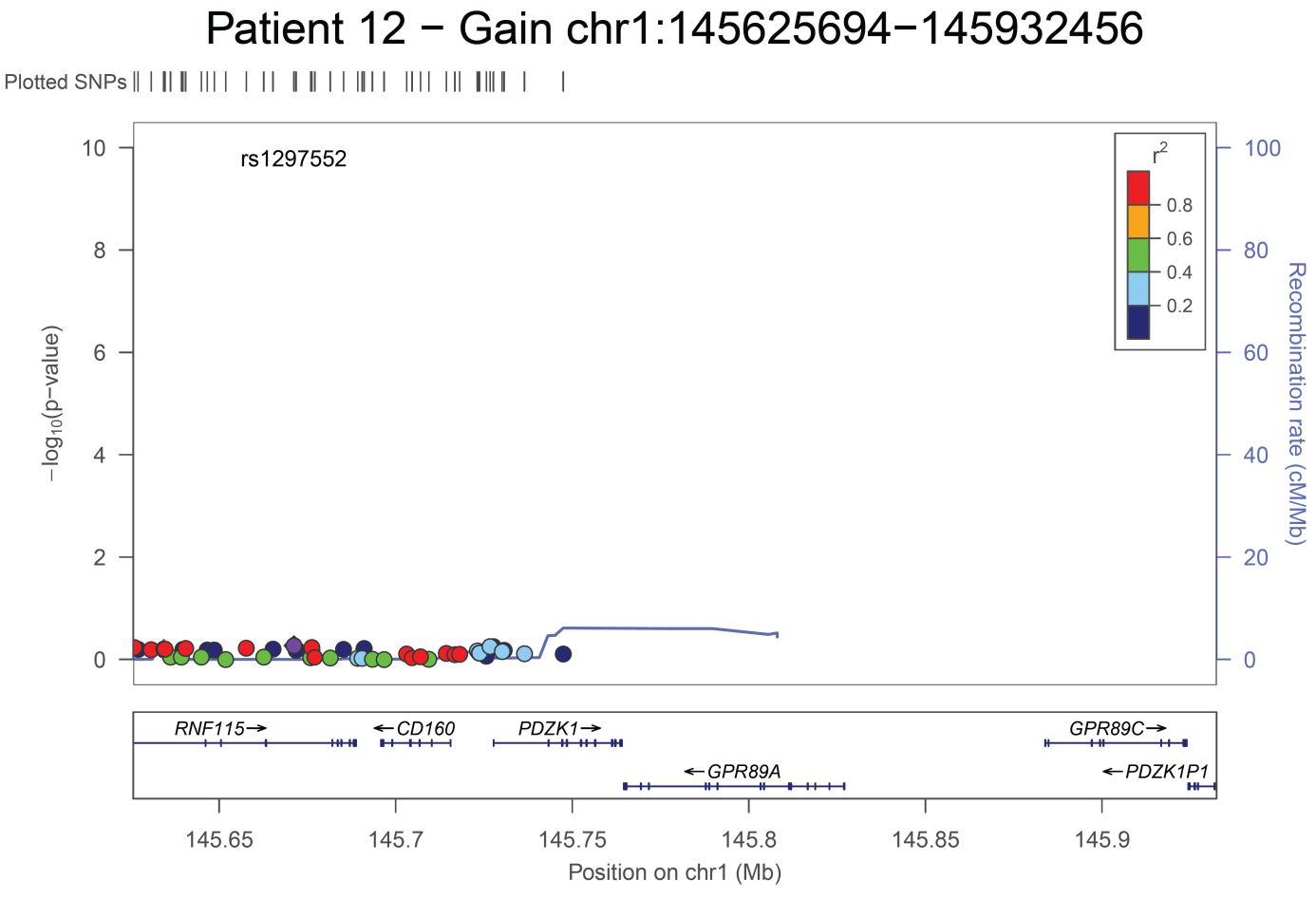


M


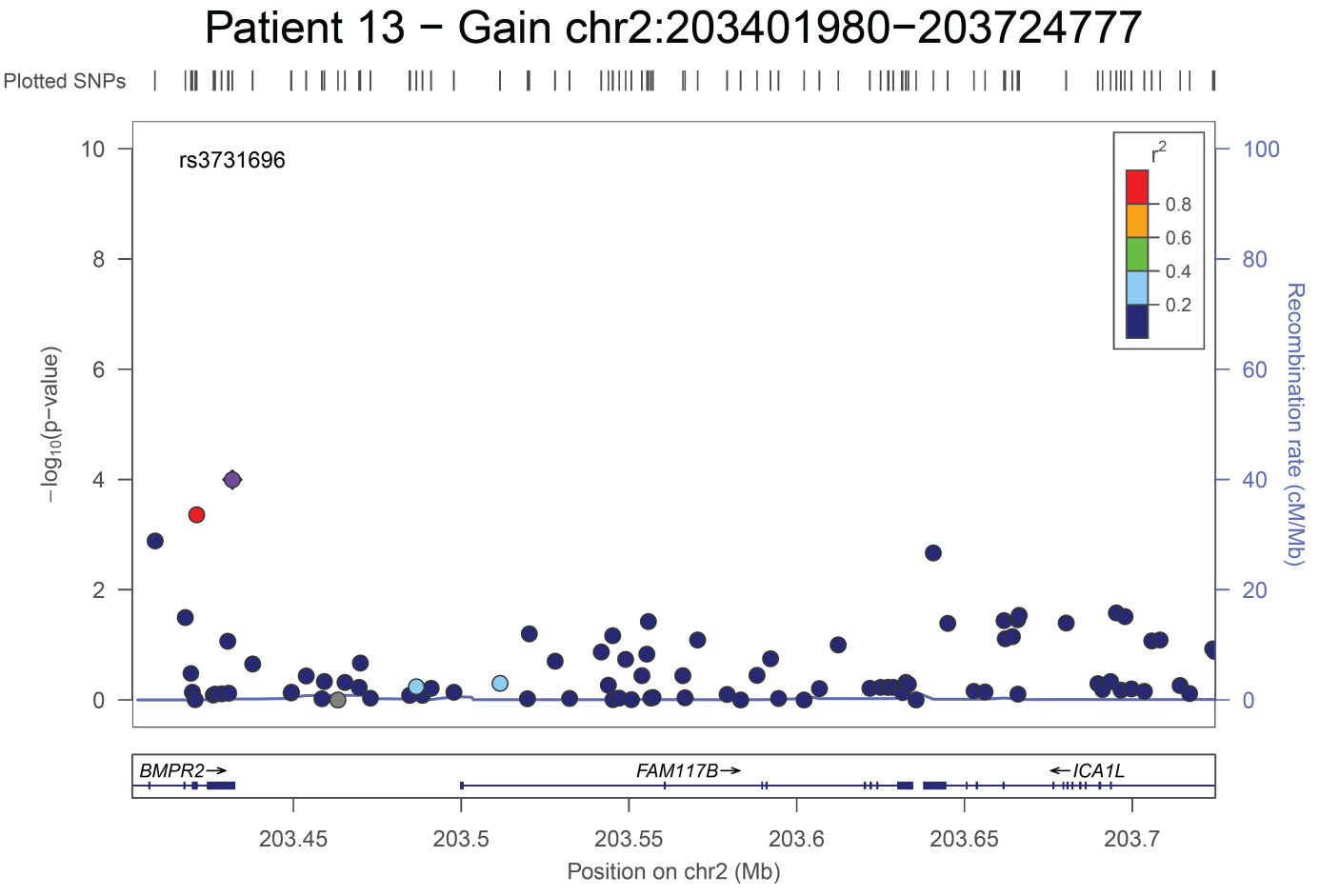


N


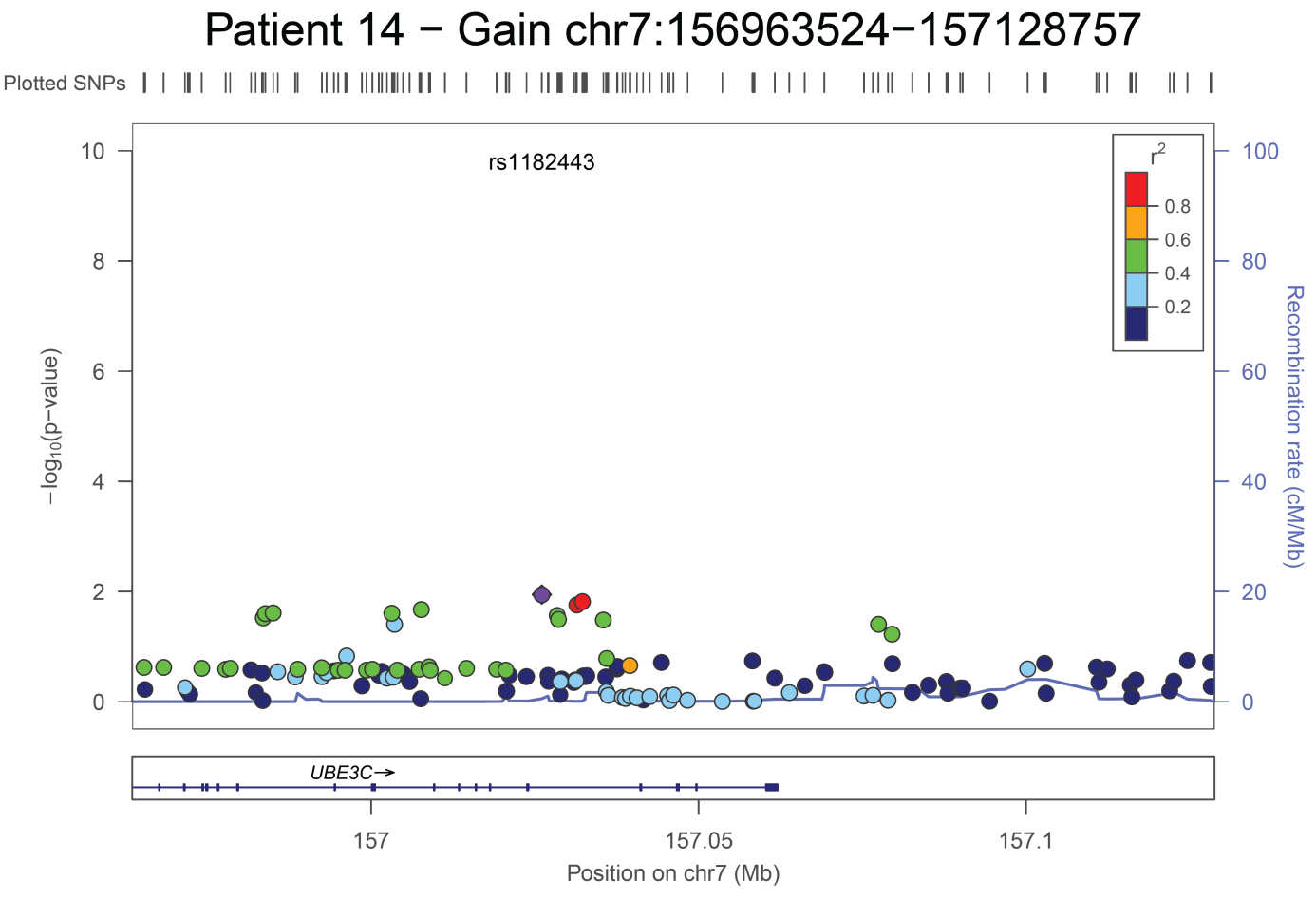


O


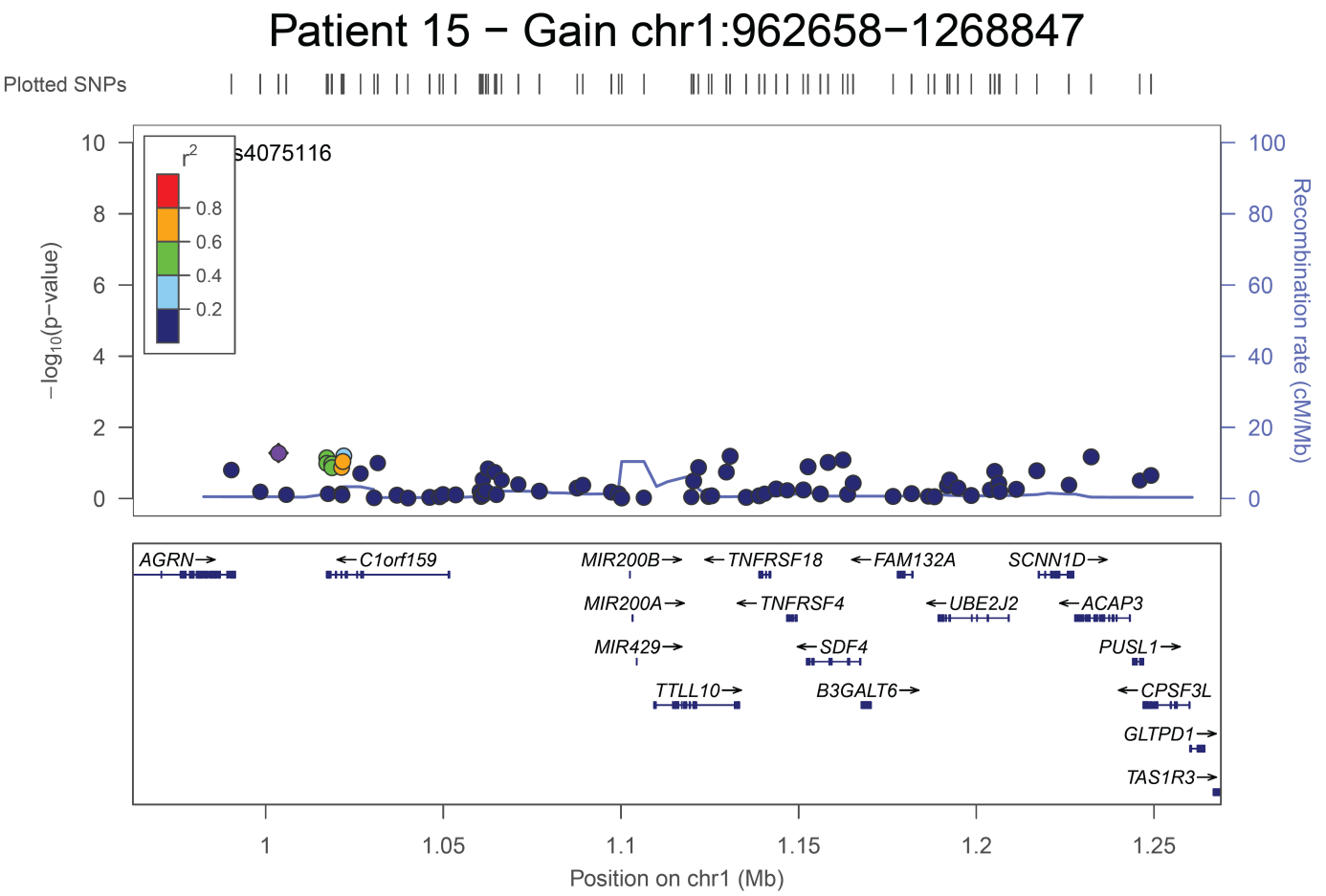


P


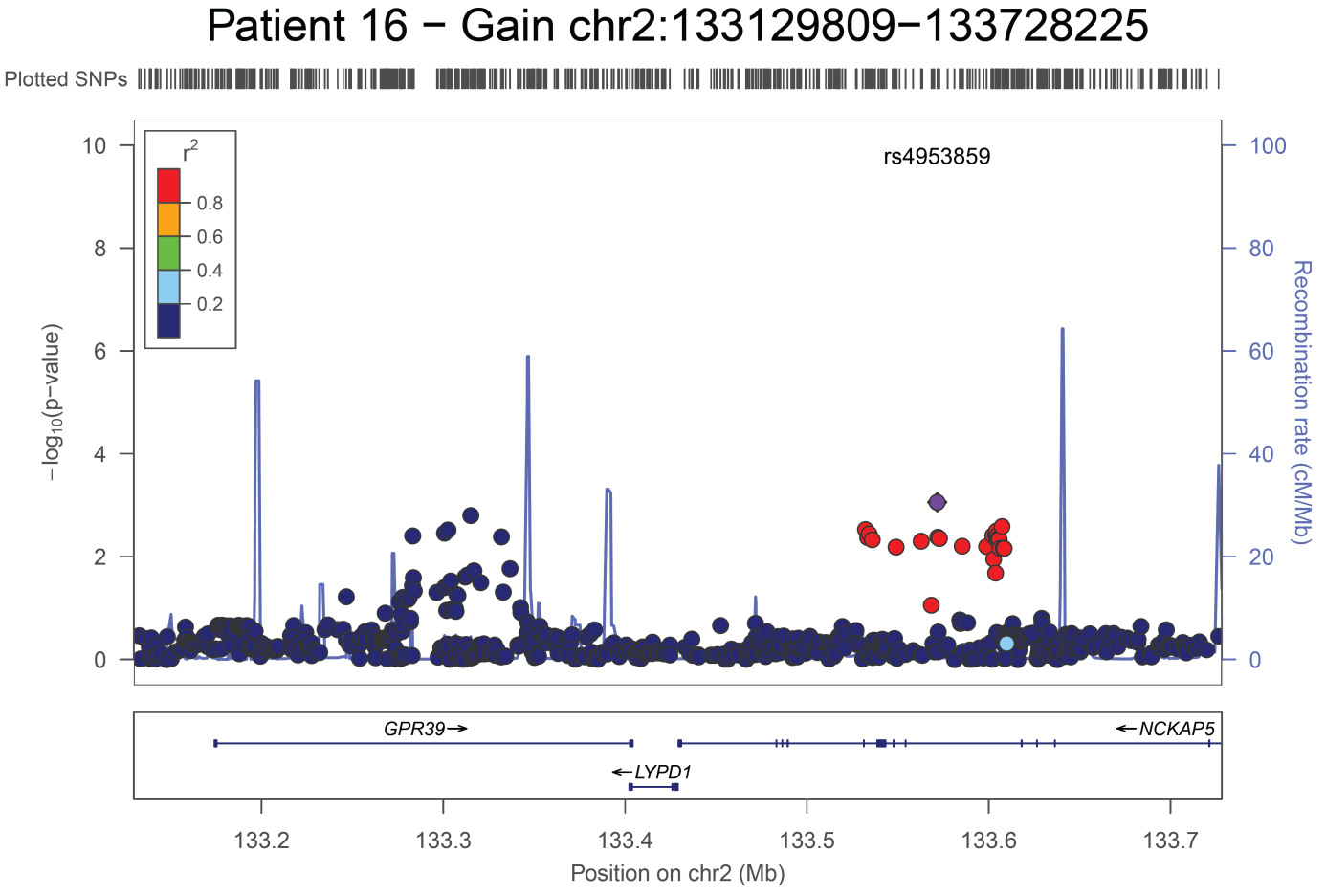


Q


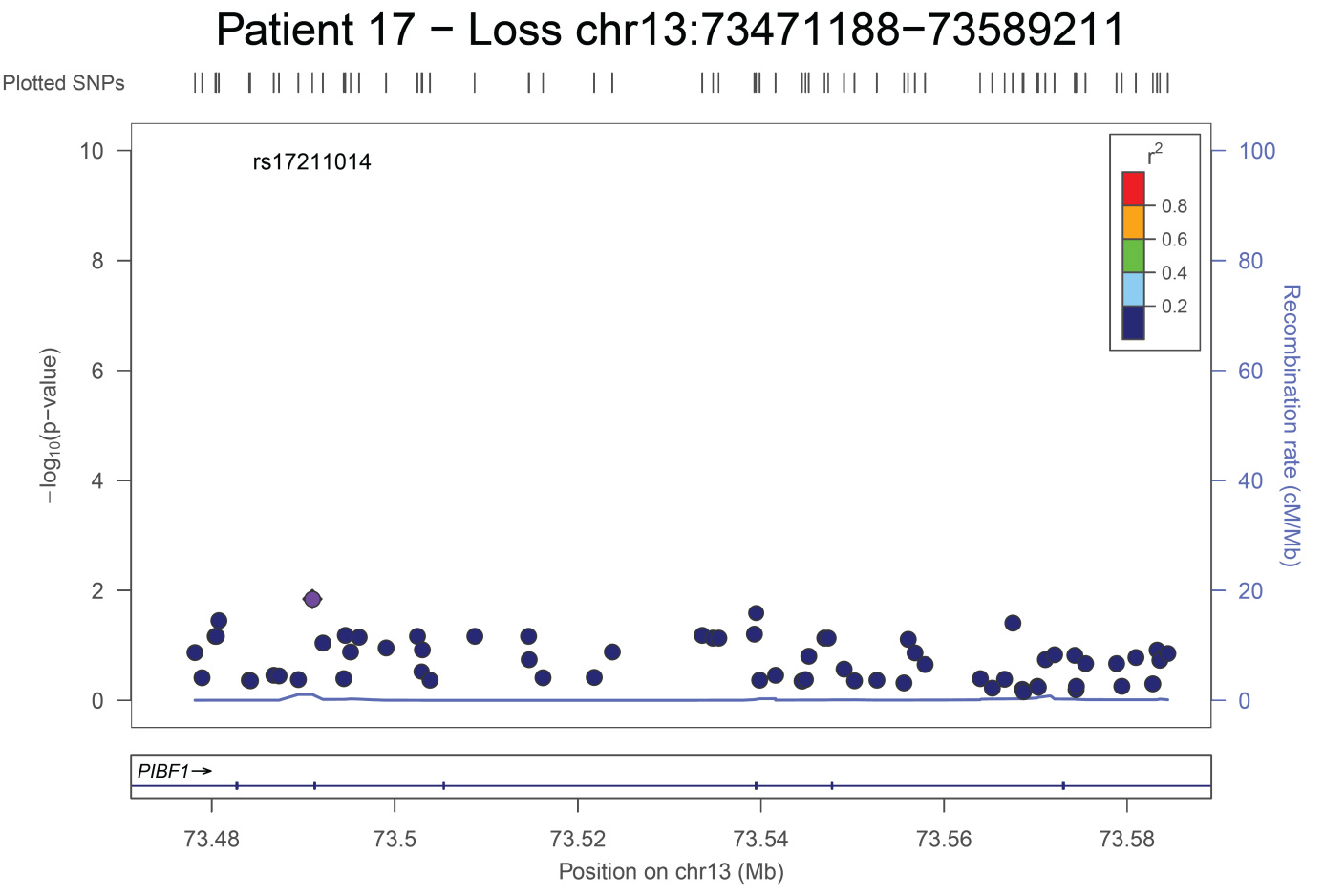


R


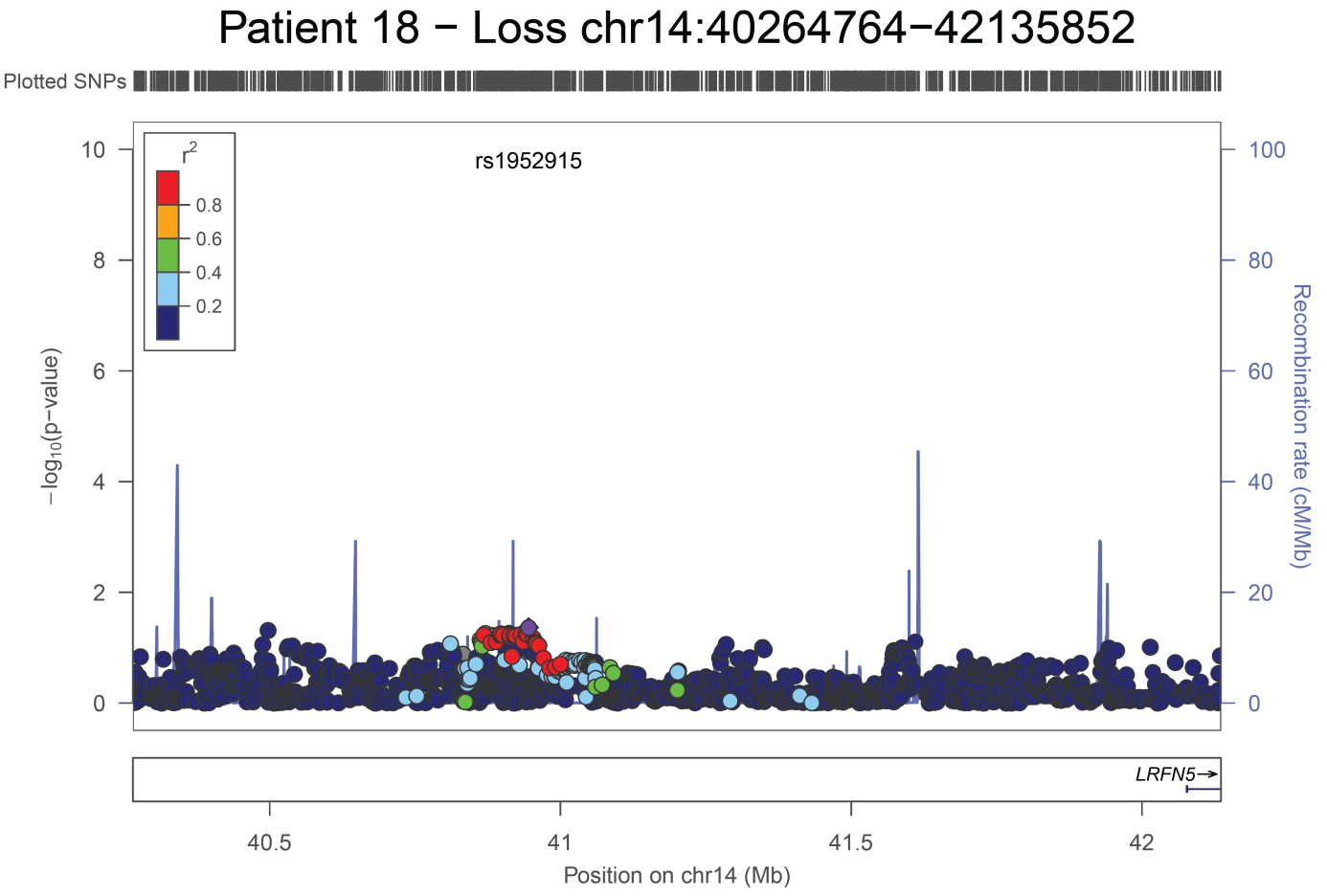


S


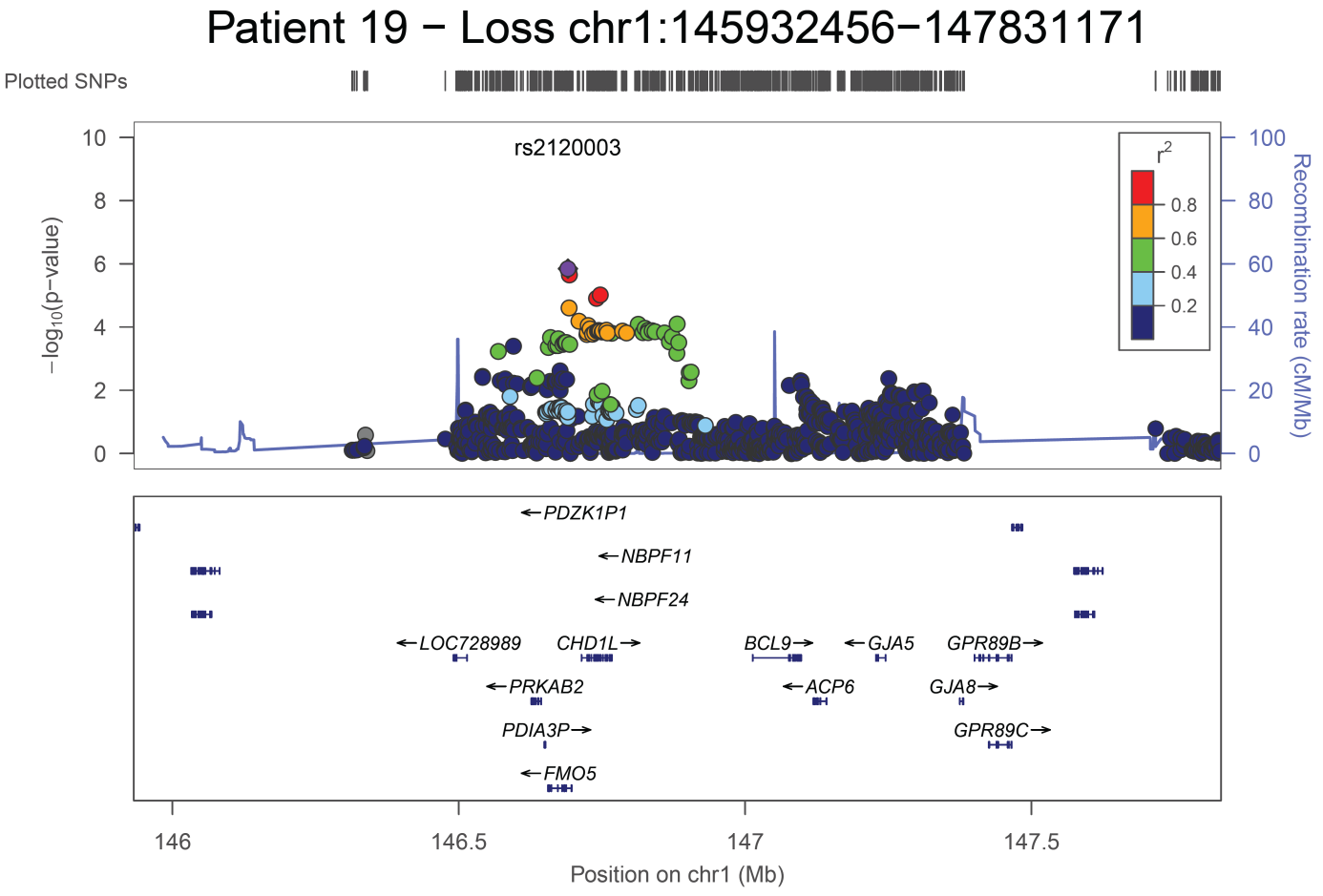


T


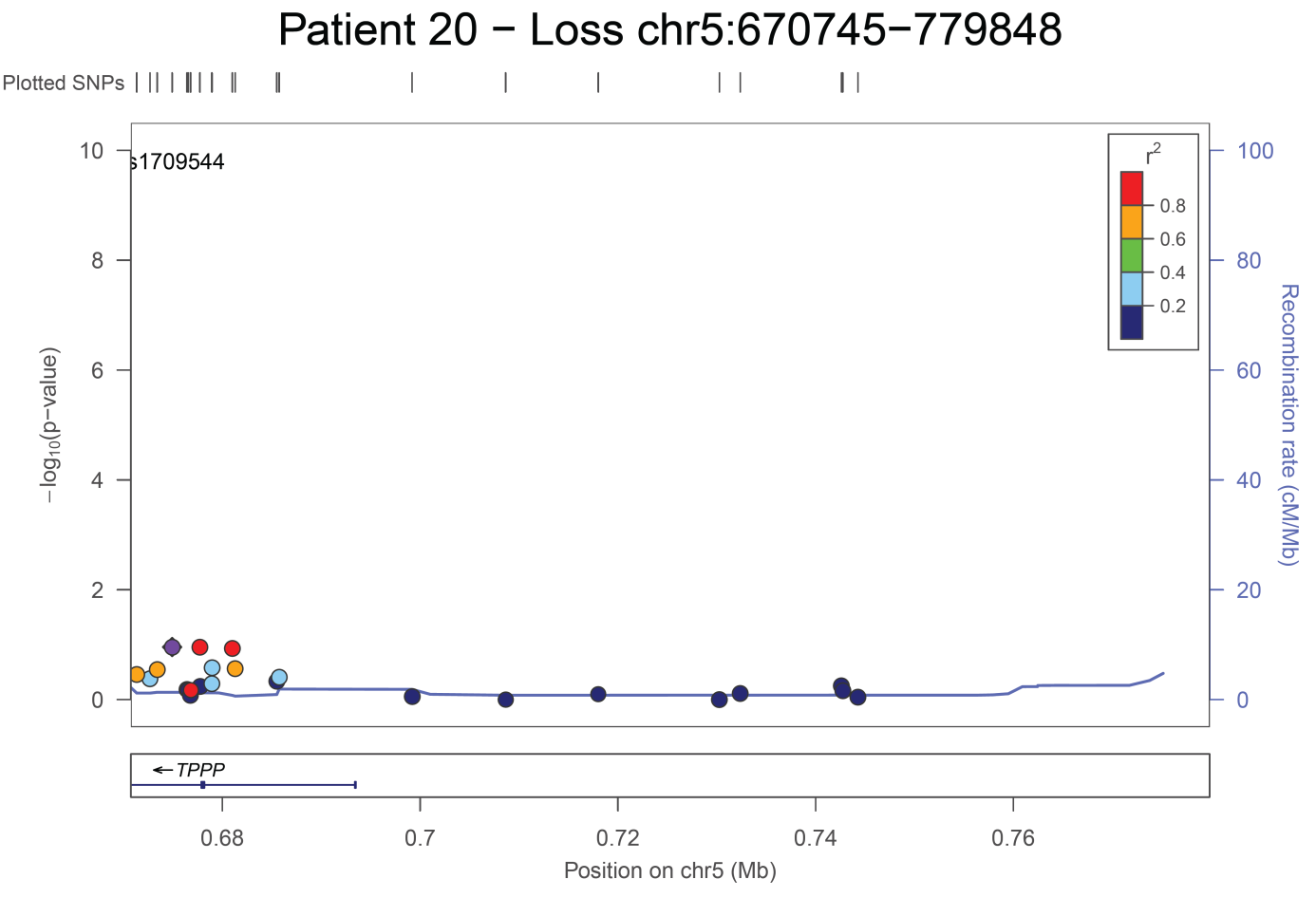

Supplement: Figure S3 — GWAS loci in the identified 20 potential causal CNVs. (A–T) The r2 values in the figure refer to the LD between the GIANT SNPs based on the CEU 1000genomes Nov 2010 samples. The blue line and right-hand y axis represent recombination rates. The SNP with the best p-value representing the best r2 value is highlighted as purple diamond. The figures were created using LocusZoom (http://csg.sph.umich.edu/locuszoom/). (DOCX) [file pgen.1003365.s003.docx]

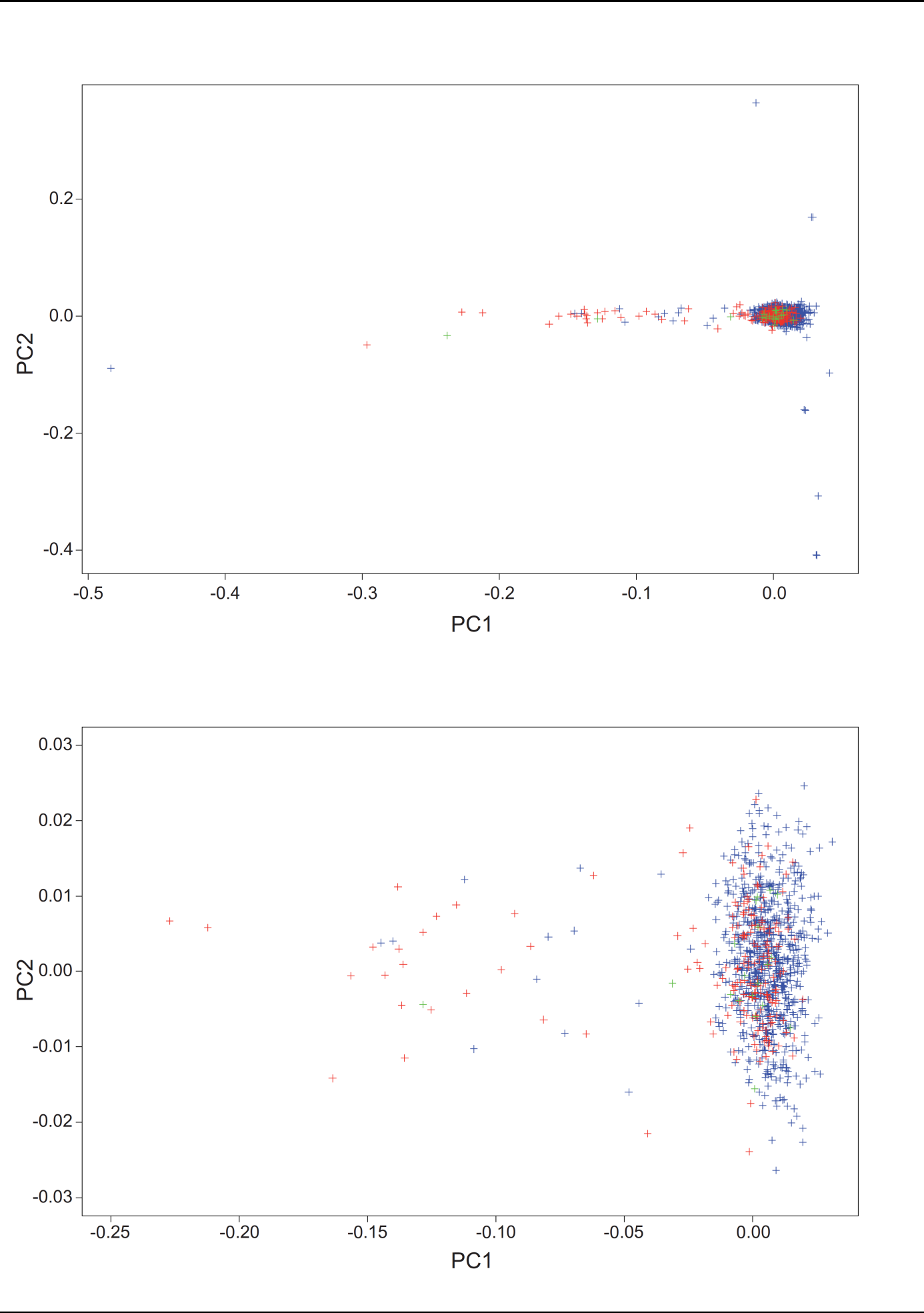

Supplement: Figure S4 — Principal component analysis of 200 patients (red and green) and 820 control individuals (blue). In the lower panel, the area containing most individuals in the upper panel has been expanded. In both groups, some samples scatter along the first principal component, owing to a residual amount of ethnic heterogeneity in both populations. Green data points indicate the patients with potentially disease-causing copy number variants. (DOCX) [file pgen.1003365.s004.docx]
