# Supplementary material for: Rare Copy Number Variants Are a Common Cause of Short Stature
Source: PLoS Genet. 2013 Mar 14;9(3):e1003365. doi: 10.1371/journal.pgen.1003365 (PMC3597495; doi:10.1371/journal.pgen.1003365)
Supplement: Figure S2 — Graphical presentation of the copy number state and MLPA confirmation. (A-T, upper pane) Presentation of the calculated copy number for the patient (green), the mother (magenta) and the father (blue) where available (Affymetrix Genotyping console). (A-T, lower pane) MLPA confirmation of one gene in each of the affected CNV regions vs. controls (C1-12). MLPAs were carried out using the SALSA MLPA Reagents kit and the P200 SALSA MLPA Reference kit according to the manufacturer's instructions (MRC Holland, Amsterdam, Netherlands). At least one MLPA probe per CNV was designed. The corresponding copy number of each locus was calculated by comparing the relative peak area of every patient to the mean peak area of the control individuals (probe ratios) with the Sequence Pilot software (JSI medical systems GmbH, Kippenheim, Germany). (DOCX) [file pgen.1003365.s002.docx]

A

B

C

D

E

F

G

H

I

J

L

K

N

M

P

O

Q

R

S

T
