# Supplementary material for: Rare Copy Number Variants Are a Common Cause of Short Stature
Source: PLoS Genet. 2013 Mar 14;9(3):e1003365. doi: 10.1371/journal.pgen.1003365 (PMC3597495; doi:10.1371/journal.pgen.1003365)
Supplement: Table S1 — MLPA probes. (DOCX) [file pgen.1003365.s005.docx]

| **Table S1. MLPA probes** | | | | | | |
| --- | --- | --- | --- | --- | --- | --- |
| Patient | Name | Sequence | Position (hg19) | Amplicon (bp) | GC (%) | Tm (° C) |
| 1 | 1q32.1_LPO | **GGGTTCCCTAAGGGTTGGA**CTGGACAGGTTACCTCCTGAGTGGAGTCA | chr1:204946107-204946164 | 100 | 55 | 76.48 |
|  | 1q32.1_RPO | TTAACTTCCCCACGTCTCAACTGAAAGGG**TCTAGATTGGATCTTGCTGGCAC** |  |  | 48 | 73.9 |
| 2 | 2q36.1-36.3_LPO | **GGGTTCCCTAAGGGTTGGA**GATCTGAAGTTGGTGTTGGAGGTGCACAAGTAA | chr2:225360611-225360676 | 108 | 45 | 76.05 |
|  | 2q36.1-36.3_RPO | CTGGCTCTAATACACGGAAGCACATATTGCAAG**TCTAGATTGGATCTTGCTGGCAC** |  |  | 45 | 74.71 |
| 3 | 14q23.1_LPO | **GGGTTCCCTAAGGGTTGGA**GATCGGACGATACGATATGTCCGATATGAATCCGAGCTA | chr14:57858303-57858380 | 120 | 46 | 78.58 |
|  | 14q23.1_RPO | CAAATGCCCGATATCATGAGACTGATCACCAAAGATCTG**TCTAGATTGGATCTTGCTGGCAC** |  |  | 44 | 78.84 |
| 4 | 22q11.21-11.22_LPO | **GGGTTCCCTAAGGGTTGGA**GGAGCCTGTCCTCACACAAAGGGCTGAACGGGGCCTGCTCAGT | chr22:22328775-22328860 | 128 | 63 | 90,64 |
|  | 22q11.21-11.22_RPO | CCACGAGTACACTGGGACCTTTGCTGGCCAGCCAGTGCGCTTC**TCTAGATTGGATCTTGCTGGCAC** |  |  | 63 | 90,14 |
| 5 | 2p23.3_LPO | **GGGTTCCCTAAGGGTTGGA**GTCGCCATGATATGCTTGCATGGGTCAAC | chr2:27245133-27245190 | 100 | 52 | 77.35 |
|  | 2p23.3_RPO | GACTCCCTGCACCTCAACTATACCAAGAT**TCTAGATTGGATCTTGCTGGCAC** |  |  | 48 | 72.23 |
| 6 | 19q13.43_LPO | **GGGTTCCCTAAGGGTTGGA**GCTGGACCCTAAATTGGATCCTCTTCCTGCTGAGA | chr19:58718103-58718172 | 112 | 51 | 80.16 |
|  | 19q13.43_RPO | GTCCCCTAATGAACATTGAGGTTGTTGAGGTCCTC**TCTAGATTGGATCTTGCTGGCAC** |  |  | 49 | 78.28 |
| 7 | 3q29_LPO | **GGGTTCCCTAAGGGTTGGA**GATCGTTCAAAGCCGTCTGAACCAATTCAACCTGTGAAT | chr3:197009589-197009666 | 120 | 44 | 79.84 |
|  | 3q29_RPO | ACTTGGGAGATTTCCAGCCTTCCAAGCTCTACTGTGACT**TCTAGATTGGATCTTGCTGGCAC** |  |  | 49 | 79. 54 |
| 8 | 1q21.1_LPO | **GGGTTCCCTAAGGGTTGGA**CAAATGCTACAGCCCCCAGGTCTTCTACCCCCTCCCATGGCCAAACT | chr1:147086292-147086385 | 136 | 57 | 89.18 |
|  | 1q21.1_RPO | ACTGCCACAGAGCCCACACCTGCTCAGAAGACTCCAGCCAAAGTGGT**TCTAGATTGGATCTTGCTGGCAC** |  |  | 57 | 88.58 |
| 9 | 22q11.22_LPO | **GGGTTCCCTAAGGGTTGGA**GGAGCCTGTCCTCACACAAAGGGCTGAACGGGGCCTGCTCAGT | chr22:22328775-22328860 | 128 | 63 | 90.64 |
|  | 22q11.22_RPO | CCACGAGTACACTGGGACCTTTGCTGGCCAGCCAGTGCGCTTC**TCTAGATTGGATCTTGCTGGCAC** |  |  | 63 | 90.14 |
| 10 | 17q11.2_LPO | **GGGTTCCCTAAGGGTTGGA**GTGCTCAAGAATATGTGGCCTGAAGGCAAAC | chr17:31065332-31065393 | 104 | 48 | 76.23 |
|  | 17q11.2_RPO | TGAGCATTACAGAGGTGACCAAGCGACCTCT**TCTAGATTGGATCTTGCTGGCAC** |  |  | 52 | 76.56 |
| 11 | 5q22.1-q23.2_LPO | **GGGTTCCCTAAGGGTTGGA**GGATTGAGTCCTGGCTGTTATGATACCTATGGT | chr5:121406219-121406284 | 108 | 45 | 74.1 |
|  | 5q22.1-q23.2_RPO | GCAGACATAGACTGCCAGTGGATTGATATTACA**TCTAGATTGGATCTTGCTGGCAC** |  |  | 42 | 72.89 |
| 12 | 1q21.1_LPO | **GGGTTCCCTAAGGGTTGGA**GACCTCCACCTTCAACCCCCGAGAATGTAAACTGTCCAAGCAA | chr1:145747046-145747131 | 128 | 51 | 84.77 |
|  | 1q21.1_RPO | GAAGGGCAAAACTATGGCTTCTTCCTGCGAATTGAGAAGGACA**TCTAGATTGGATCTTGCTGGCAC** |  |  | 47 | 82.31 |
| 13 | 2q33.2_LPO | **GGGTTCCCTAAGGGTTGGA**GCAACCAAGACATGTCAGCCTGGTTCAATCTGTTT | chr2:203644290-203644359 | 112 | 46 | 78.53 |
|  | 2q33.2_RPO | GCAGACTTGGATCCACTTTCAAACCCAGATGCTAT**TCTAGATTGGATCTTGCTGGCAC** |  |  | 46 | 77.46 |
| 14 | 7q36.3_LPO | **GGGTTCCCTAAGGGTTGGA**GATAATGTTGTCTCGATGCCTTCGAGATGCATG | chr7:157000446-157000511 | 108 | 45 | 76.27 |
|  | 7q36.3_RPO | CCTGGGGATCATCAAGTTGGCTTATCCAGAAAC**TCTAGATTGGATCTTGCTGGCAC** |  |  | 48 | 77.78 |
| 15 | 1p36.33_LPO | **GGGTTCCCTAAGGGTTGGA**GACAACTGGCAGTGCAGAGTTTAGCATTTAATTTGAAAG | chr1:1191424-1191501 | 120 | 38 | 75.42 |
|  | 1p36.33_RPO | ATAAAGTCTTTTGTGAATTATTTCCTGAAGTCGTGGAGG**TCTAGATTGGATCTTGCTGGCAC** |  |  | 36 | 73.40 |
| 16 | 2q21.2_LPO | **GGGTTCCCTAAGGGTTGGA**CACGTTGATGCGGACGATGACCCTTCCACCTTAGC | chr2:133542892-133542961 | 112 | 57 | 83.65 |
|  | 2q21.2_RPO | ATTGCTCCAAGCAGTTCCAAACCAGAGCTGCAGGC**TCTAGATTGGATCTTGCTGGCAC** |  |  | 54 | 82.00 |
| 17 | 13q22.1_LPO | **GGGTTCCCTAAGGGTTGGA**CGTCAGAGAGATTCTAAGATTGATTCACTGACGGAAT | chr13:73539478-73539551 | 116 | 41 | 74.87 |
|  | 13q22.1_RPO | CTATTGCACAACTTGAGAAAGATGTCAGGTAAACCAT**TCTAGATTGGATCTTGCTGGCAC** |  |  | 38 | 73.75 |
| 18 | 14q21.1-q21.2_LPO | **GGGTTCCCTAAGGGTTGGA**CAGAAGCTTGGAAAGCGCCAATATTAACCTGCTTCCAGGTACATCAC | chr14:42076938-42077031 | 136 | 47 | 82.63 |
|  | 14q21.1-q21.2_RPO | AAGGTCAAGAGAGTGTCTTGCAGCCCTCTTGTTTCCGTGTATCTACA**TCTAGATTGGATCTTGCTGGCAC** |  |  | 47 | 81.9 |
| 19 | 1q21.1_LPO | **GGGTTCCCTAAGGGTTGGA**CAAATGCTACAGCCCCCAGGTCTTCTACCCCCTCCCATGGCCAAACT | chr1:147086292-147086385 | 136 | 57 | 89.18 |
|  | 1q21.1_RPO | ACTGCCACAGAGCCCACACCTGCTCAGAAGACTCCAGCCAAAGTGGT**TCTAGATTGGATCTTGCTGGCAC** |  |  | 57 | 88.58 |
| Underlined letters mark specific target sequences, bold letters are universal primer sequences. Tm and GC content were calculated in regards of the specific target sequence. For all oligonucleotides a melting temperature of ≥ 70°C and a GC content of about 40-60 % was used. | | | | | | |
