# Supplementary material for: Rare Copy Number Variants Are a Common Cause of Short Stature
Source: PLoS Genet. 2013 Mar 14;9(3):e1003365. doi: 10.1371/journal.pgen.1003365 (PMC3597495; doi:10.1371/journal.pgen.1003365)
Supplement: Table S2 — Murine knock-out phenotypes of the Mouse Genome Informatics database (MGI) and Haploinsufficiency scores of genes within the identified CNVs. (DOCX) [file pgen.1003365.s006.docx]

| **Table S2. Murine knock-out phenotypes of the Mouse Genome Informatics database (MGI) and Haploinsufficiency scores of genes within the identified CNVs** | | | | | | | |  |
| --- | --- | --- | --- | --- | --- | --- | --- | --- |
|  |  |  | **Mouse genome Database** | |  | **Haploinsufficiency score (<10%)** | |  |
| Patient | Loss / Gain | Band | # of genes | genes |  | # of genes | genes | |
| 1 | Loss | 1q32.1 | 3 | PPP1R15B, MDM4, NFASC |  | 3 | MDM4 (5.4%), ELK4 (1.1%), NUCKS1 (4.6%) | |
| 2 | Loss | 2q36.1-36.3 | 4 | PAX3, ACSL3, CUL3, IRS |  | 5 | EPHA4 (7.3%), PAX3 (0.6%), ACSL3 (6.5%), SCG2 (6.5%), IRS1 (2.3%) | |
| 3 | Loss | 14q23.1 | 1 | OTX2 |  | 3 | OTX2 (3.4%), EXOC5 (7.5%), C14orf105 (9.2) | |
| 4 | Loss | 22q11.21-11.22 | 2 | UBE2L3, MAPK1 |  | 3 | UBE2L3 (5.7%), MAPK1 (0.2%), PPM1F (1.9%) | |
| 5 | Gain | 2p23.3 | 7 | POMC, DNMT3A, ASXL2, HADHA, CENPA, IFT172, FOSL2 |  | 8 | DNMT3A (5.2%), CENPA (9.9%), MAPRE3 (3.9%), CAD (8.7%), EIF2B4 (8.1%), PPM1G (0.3%), FOSL2 (5.0%), PPP1CB (8.6%) | |
| 6 | Gain | 19q13.43 | 1 | ZNF274 |  | 0 | - | |
| 7 | Gain | 3q29 | 5 | TFRC, OSTalpha, PCYT1A, PAK2, DLG1 |  | 3 | PCYT1A (8.2%), PAK2 (6.7%), DLG1 (0.7) | |
| 8 | Loss | 1q21.1 | 0 | - |  | 2 | PRKAB2 (9.7%), BCL9 (7.1) | |
| 9 | Loss | 22q11.22 | 0 | - |  | 0 | - | |
| 10 | Gain | 17q11.2 | 0 | - |  | 0 | - | |
| 11 | Gain | 5q22.1-q23.2 | 7 | STARD4, APC, KCNN2, PGGT1B, CDO1, HSD17B4, SNX2 |  | 2 | MCC (9.4%), KCNN2 (6.8%) | |
| 12 | Gain | 1q21.1 | 0 | - |  | 0 | - | |
| 13 | Gain | 2q33.2 | 1 | BMPR2 |  | 1 | BMPR2 (4.5%) | |
| 14 | Gain | 7q36.3 | 0 | - |  | 0 | - | |
| 15 | Gain | 1p36.33 | 1 | AGRN |  | 1 | SCNN1D (7.9%) | |
| 16 | Gain | 2q21.2 | 0 | - |  | 0 | - | |
| 17 | Loss | 13q22.1 | 0 | - |  | 0 | - | |
| 18 | Loss | 14q21.1-q21.2 | 0 | - |  | 0 | - | |
| 19 | Loss | 1q21.1 | 0 | - |  | 2 | PRKAB2 (9.7%), BCL9 (7.1) | |
| 20 | Loss | 5p15.33 | 0 | - |  | 3 | MDM4 (5.4%), ELK4 (1.1%), NUCKS1 (4.6%) | |
