# Supplementary material for: Rare Copy Number Variants Are a Common Cause of Short Stature
Source: PLoS Genet. 2013 Mar 14;9(3):e1003365. doi: 10.1371/journal.pgen.1003365 (PMC3597495; doi:10.1371/journal.pgen.1003365)
Supplement: Table S3 — Candidate genes based on their function. (DOCX) [file pgen.1003365.s007.docx]

| **Table S3. Candidate genes based on their function** | | |  |
| --- | --- | --- | --- |
| **Gene Symbol** | **Gene description** | **known or assumed gene function** | **# of controls with CNVs affecting this gene^1^** |
| PATIENT 1 – 1q32.1 – LOSS – DE NOVO | | |  |
| DSTYK | dual serine/threonine and tyrosine protein kinase | Presumed regulator of cell death | 0 |
| MDM4 | Mdm4 p53 binding protein homolog (mouse) | Inhibits cell-cycle arrest, regulates apoptosis | 0 |
| NUCKS1 | nuclear casein kinase and cyclin-dependent kinase substrate 1 | May play a role in mitosis | 0 |
| PIK3C2B | phosphoinositide-3-kinase, class 2, beta polypeptide | Plays a role cell proliferation, oncogenic transformation, cell survival, cell migration, and intracellular protein trafficking | 0 |
| RBBP5 | retinoblastoma binding protein 5 | Regulates cell proliferation, plays role in epigenetic transcriptional activation | 0 |
| PATIENT 2 – 2q36.1-36.3 – LOSS – DE NOVO | | |  |
| CUL3 | cullin-3 | Involved in ubiquitinylation and cell cycle regulation | 0 |
| IRS1 | insulin receptor substrate 1 | May mediate the control of various cellular processes by insulin | 0 |
| PAX3 | paired box 3 | Plays critical role during fetal development | 0 |
| PATIENT 3 – 14q23.1 – LOSS – DE NOVO | | |  |
| AP5M1 | AP5M1 adaptor-related protein complex 5, mu 1 subunit | May play role in cell death and endosomal transport | 0 |
| PATIENT 4 – 22q11.21-11.22 – LOSS – DE NOVO | | |  |
| MAPK1 | Mitogen-activated protein kinase 1 | Different cellular processes like proliferation, signaling etc. | 0 |
| TOP3B | Topoisomerase (DNA) III beta | Plays role in DNA topology, recombination, aging and genome stability | 1 |
| YPEL1 | Yippee-like 1 (Drosophila) | May play role in the regulation of cellular morphology and during craniofacial development | 0 |
| ZNF280A | Zinc finger protein 280A | May be involved in transcriptional regulation | 0 |
| ZNF280B | Zinc finger protein 280B | May be involved in transcriptional regulation | 0 |
| PATIENT 5 – 2p23.3 – GAIN – DE NOVO | | |  |
| AGBL5 | ATP/GTP binding protein-like 4 | Mediates tubulin processing | 0 |
| ASXL2 | ASXL2 additional sex combs like 2 | Putative polycomb gene, may regulate homeotic genes during development | 0 |
| CENPA | centromere protein A | Required for recruitment and assembly of kinetochore proteins, mitotic progression and chromosome segregation | 0 |
| DNMT3A | DNA (cytosine-5-)-methyltransferase 3 alpha | Functions in de novo methylation which is important for embryonic development, imprinting, and X-chromosome inactivation | 0 |
| FOSL2 | FOS-like antigen 2 | Controls osteoclast survival and size | 0 |
| GPN1 | GPN-loop GTPase 1 | May play role in DNA repair and activation of transcription | 0 |
| GTF3C2 | general transcription factor IIIC, polypeptide 2, beta 110kDa | Required for RNA polymerase III-mediated transcription | 0 |
| IFT172 | intraflagellar transport 172 homolog (Chlamydomonas) | Required for the maintenance and formation of cilia | 0 |
| MAPRE3 | microtubule-associated protein, RP/EB family, member 3 | Promotes microtubule growth, may be involved in cytokinesis and cell migration | 0 |
| PPP1CB | protein phosphatase 1, catalytic subunit, beta isozyme | Subunit of protein phosphatase 1, involved in cell division, glycogen metabolism, muscle contractility, protein synthesis | 0 |
| SUPT7L | suppressor of Ty 7 (S. cerevisiae)-like | Subunit of a chromatin-remodelling-complex | 0 |
| ZNF512 | zinc finger protein 512 | May be involved in transcriptional regulation | 0 |
| ZNF513 | zinc finger protein 513 | May be involved in transcriptional regulation | 0 |
| PATIENT 6 – 19q13.43 – GAIN – DE NOVO | | |  |
| ZNF135 | zinc finger protein 135 | May be involved in transcriptional regulation | 0 |
| ZNF256 | zinc finger protein 256 | Transcriptional repressor that plays a role in cell proliferation | 0 |
| ZNF274 | zinc finger protein 274 | Probable transcription repressor | 0 |
| ZNF329 | zinc finger protein 329 | May be involved in transcriptional regulation | 0 |
| ZNF417 | zinc finger protein 417 | May be involved in transcriptional regulation | 0 |
| ZNF418 | zinc finger protein 418 | Transcriptional repressor | 0 |
| ZNF544 | zinc finger protein 544 | May be involved in transcriptional regulation | 0 |
| ZNF606 | zinc finger protein 606 | Probable transcription repressor | 0 |
| ZSCAN1 | zinc finger and SCAN domain containing 1 | May be involved in transcriptional regulation | 0 |
| ZSCAN18 | zinc finger and SCAN domain containing 18 | May be involved in transcriptional regulation | 0 |
| PATIENT 7 – 3q29 – GAIN – DE NOVO | | |  |
| CEP19 | centrosomal protein 19kDa | Localizes to spindle pole during mitosis | 0 |
| DLG1 | discs, large homolog 1 | Essential multidomain scaffolding protein required for normal development | 0 |
| PAK2 | p21 protein (Cdc42/Rac)-activated kinase 2 | Plays role in cytoskeleton regulation, cell motility, cell cycle progression, apoptosis and proliferation | 0 |
| RNF168 | ring finger protein 168 | Involved in DNA repair | 0 |
| UBXN7 | UBX domain-containing protein 7 | May be involved in DNA repair | 0 |
| PATIENT 8 – 1q21.1 – LOSS – MATERNALLY INHERITED | | |  |
| BCL9 | B-cell CLL/lymphoma 9 | Involved in signal transduction through the Wnt pathway | 0 |
| CHD1L | chromodomain helicase DNA binding protein 1-like | Plays role in chromatin-remodeling following DNA damage | 0 |
| PRKAB2 | protein kinase, AMP-activated, beta 2 non-catalytic subunit | Regulatory unit of AMPK, plays role in cell growth and proliferation | 0 |
| PATIENT 9 – 22q11.22 – LOSS – MATERNALLY INHERITED | | |  |
| TOP3B | topoisomerase (DNA) III beta | Plays role in DNA topology, recombination, aging and genome stability | 1 |
| PATIENT 11 – 5q22.1-q23.2 – GAIN – MATERNALLY INHERITED | | |  |
| APC | adenomatous polyposis coli | Tumor suppressor, participates in Wnt signaling | 0 |
| CAMK4 | calcium/calmodulin-dependent protein kinase IV | May be involved in transcriptional regulation | 0 |
| CEP120 | centrosomal protein 120kDa | Functions in the microtubule-dependent coupling of the nucleus and the centrosome | 0 |
| CSNK1G3 | casein kinase 1, gamma 3 | Participates in Wnt pathway | 0 |
| EPB41L4A | erythrocyte membrane protein band 4.1 like 4A | May regulate the interaction between cytoskeleton and plasma membrane | 0 |
| LOX | lysyl oxidase | Initiates the crosslinking of collagens and elastin | 0 |
| MCC | mutated in colorectal cancers | May negatively regulate cell cycle progression | 0 |
| PRDM6 | PR domain containing 6 | May participate in chromatin remodelling | 0 |
| SEMA6A | sema domain, transmembrane domain (TM), and cytoplasmic domain, (semaphorin) 6A | Promotes reorganization of the actin cytoskeleton | 0 |
| SRFBP1 | serum response factor binding protein 1 | May be involved in regulating transcriptional activation | 0 |
| WDR36 | WD repeat domain 36 | Members of the WD repeat protein family are involved in a variety of cellular processes, including cell cycle progression, signal transduction, apoptosis, and gene regulation | 5 |
| ZNF608 | zinc finger protein 608 | May be involved in transcriptional regulation | 0 |
| PATIENT 13 – 2q33.2 – GAIN – MATERNALLY INHERITED | | |  |
| BMPR2 | Bone morphogenetic protein receptor, type II (serine/threonine kinase) | Involved in endochondral bone formation and embryogenesis | 0 |
| PATIENT 15 – 1p36.33 – GAIN – MATERNALLY INHERITED | | |  |
| CPSF3L | Cleavage and polyadenylation specific factor 3-like | Processes small RNAs | 8 |
| PATIENT 19 – 1q21.1 – LOSS – PATERNALLY INHERITED | | |  |
| BCL9 | B-cell CLL/lymphoma 9 | Involved in signal transduction through the Wnt pathway | 0 |
| CHD1L | chromodomain helicase DNA binding protein 1-like | Plays role in chromatin-remodeling following DNA damage | 0 |
| PRKAB2 | protein kinase, AMP-activated, beta 2 non-catalytic subunit | Regulatory unit of AMPK, plays role in cell growth and proliferation | 0 |
| PATIENT 20 – 5p15.33 – LOSS – PATERNALLY INHERITED | | |  |
| TPPP | tubulin polymerization-promoting protein | May play role in microtubule polymerization and mitotic spindle assembly | 0 |

^1^ **No. of controls with correspondent CNVs (deletion or duplication) affecting this gene**
