# Supplementary material for: Rare Copy Number Variants Are a Common Cause of Short Stature
Source: PLoS Genet. 2013 Mar 14;9(3):e1003365. doi: 10.1371/journal.pgen.1003365 (PMC3597495; doi:10.1371/journal.pgen.1003365)
Supplement: Table S4 — Detailed clinical data of patients with the identified 20 CNVs. (DOCX) [file pgen.1003365.s008.docx]

| **Table S4. Detailed clinical data of patients with the identified 20 CNVs** | | | | | | | | | | | | | | | | |
| --- | --- | --- | --- | --- | --- | --- | --- | --- | --- | --- | --- | --- | --- | --- | --- | --- |
| Patient | Gender | Ethnicity | Proportions | isolated | Prenatal anomalies | Weeks of gestation | Birth length [cm/SDS] | Birth weight [g/SDS] | OFC at birth [cm/SDS] | Age at investigation [y] | Height [cm/SDS] | OFC [cm/SDS] | Facial gestalt | Behavi-oural anomalies | | Others |
| 1 | female | European | proportionate | - | - | 41 | 53/0,36 | 2600/-2,28 | 36/0,62 | 16 | 147/-2,96 | 48,8/-3,57 | assymetry, retrognathia, | learning disability | |  |
| 2 | male | European | proportionate | + | - | 40 | 50/-1,09 | 2950/-1,53 | 33,5/-1,62 | 3 | 92,5/-2,98 | 47,6/-2,84 | wide spaced eyes, small mouth, broad nasal tip | - | | dystrophy |
| 3 | male | European | proportionate | - | - | 39 | 51/-0,39 | 3590/0,24 | 37,5/1,69 |  | 62/-4,43 | 44,2/-1,25 | micropthalmia | learning disability | | persistent ductus arteriosus |
| 4 | male | European | proportionate | - | - | 36 | 45/-1,58 | 2200/-1,49 | 32/-1,13 | 2 | 71/-6,95 | n.a. | low set ears, retrognathia, long philtrum | mild delay | |  |
| 5 | female | European | proportionate | + | + | 39 | 46/-2,32 | 2750/-1,42 | 35/0,31 | 7 | 111,1/-3,57 | 50/-1,34 | - | - | | Syndactyly II/III of toes |
| 6 | male | European | proportionate | - | + | 40 | 48/-1,96 | 2690/-2,12 | 31/-3,54 | 5 | 102/-3,16 | 47,6/-3,48 | - | Ptosis | | preaxial polydaytyly |
| 7 | female | European | proportionate | + | - | 38 | 48/-1,04 | 2990/-0,44 | 35/0,54 | 12 | 137,1/-2,82 | 55/1,53 | - | - | | - |
| 8 | female | European | short extremities | + | + | 40 | 48/-1,96 | 3180/-1,01 | 33/-2 | 4 | 92,3/-3,03 | 48,1/-2,47 | retrognathia | - | | brachydactyly |
| 9 | female | Asian | proportionate | + | - | 39 | 48/-1,41 | 2740/-1,44 | 32,5/-1,62 | 4 | 90,4/-3,39 | 46/-3,23 | prominent forehead, low set ears | - | | brachydactyly |
| 10 | female | European | proportionate | - | - | 38 | 49/-0,61 | 2790/-0,91 | 31,5/-2,15 | 1 | 78/-1,2 | 44,5/-2,28 | retrognathia, prominent nose, preauricular tags, sparse hair | | - | convex nails |
| 11 | female | European | proportionate | - | + | 38 | 44/-2,78 | 2370/-1,9 | n.a. | 9 | 116,85/-3,48 | 46,2/-4,39 | - | learning disability | | - |
| 12 | male | European | short extremities | - | + | 36 | 42/-2,73 | 2100/-1,71 | 33,5/-0,19 | 9 | 120/-2,55 | 54/0,65 | asymmetry, low set ears, short neck | - | | muscular hypotonia |
| 13 | male | European | proportionate | + | - | 40 | 50/-1,09 | 3040/-1,32 | 34/-1,23 | 10 | 120/-3,24 | 55,5/1,63 | - | - | | - |
| 14 | male | European | short trunk | + | - | 38 | 51/0,26 | 3270/0,22 | 32,5/-1,38 | 4 | 95,5/-2,01 | 47/-2,46 | - | - | | - |
| 15 | male | European | short extremities | + | - | 38 | 52/0,39 | 3650/0,78 | 35/0,07 | 8 | 114,5/-3,47 | 52/-0,8 | broad nose | - | | - |
| 16 | female | European | proportionate | + | - | 36 | 47/-0,54 | 3090/0,77 | 34,5/0,8 | 3 | 91,5/-3,09 | 48/-1,69 | - | - | | - |
| 17 | female | European | proportionate | + | + | 41 | 47/-2,36 | 3640/0,14 | 37/1,38 | 3 | 88/-4,04 | 47/-2,46 | - | - | | - |
| 18 | female | European | proportionate | + | - | 27 | 32/-0,97 | 670/-1,21 | n.a. | 9 | 114,2/-3,96 | 44,5/-5,67 | - | - | | - |
| 19 | female | European | proportionate | - | + | 37 | 42/-3,13 | 1600/-3,16 | 28/-4,21 | 4 | 92,1/-2,93 | 45,4/-3,69 | frontal bossing, bulbous nasal tip, | language delay | | Hexadactyly, sakral dimple, sparse hair |
| 20 | male | European | proportionate | + | + | 40 | 48/-1,96 | 3110/-1,17 | 34/-1,23 | 4 | 90,1/-3,56 | 48/-2,54 | - | - | | - |
