# Supplementary material for: Rare Copy Number Variants Are a Common Cause of Short Stature
Source: PLoS Genet. 2013 Mar 14;9(3):e1003365. doi: 10.1371/journal.pgen.1003365 (PMC3597495; doi:10.1371/journal.pgen.1003365)
Supplement: Table S5 — Association results for the identified CNVs from genome wide association of human height variation GIANT consortium data. Signals with p values<1.377×10^−6^ are marked (*). (DOCX) [file pgen.1003365.s009.docx]

| **Table S5. Association results for the identified CNVs from genome wide association of human height variation (Lango Allen et al. 2010) GIANT consortium data. Signals with *P* values <1.377x10^-6^ are marked *.** | | | | | | | | | | |
| --- | --- | --- | --- | --- | --- | --- | --- | --- | --- | --- |
|  |  |  |  |  |  | **GWAS loci** | | | | |
| Patient | Inheritance | Loss / Gain | Position | Size(kb) | #Genes | # SNPs in CNV region | max P value | min P value | min P value reference SNP | min P value gene region / flanking genes |
| 1 | de novo | Loss | 1q32.1 | 2157 | 33 | 1898 | 1 | 4.5 x 10^−4^ | rs951366 | NUCKS1 3' UTR |
| 2 | de novo | Loss | 2q36.1-36.3 | 6695 | 31 | 6440 | 1 | 2.17 x 10^−8*^ | rs2602675 | SERPINE2 |
| 3 | de novo | Loss | 14q23.1 | 1352 | 8 | 1409 | 1 | 1.19 x 10^−2^ | rs7154287 | SLC35F4 |
| 4 | de novo | Loss | 22q11.21-11.22 | 1363 | 23 | 919 | 9.95 x 10^−1^ | 1.1 x 10^−3^ | rs5754217 | UBE2L3 |
| 5 | de novo | Gain | 2p23.3 | 4573 | 77 | 3354 | 1 | 2.79 x 10^−13*^ | rs11125884 | EFR3B promoter |
| 6 | de novo | Gain | 19q13.43 | 367 | 13 | 253 | 1 | 1.26 x 10^−1^ | rs1469087 | ZNF135 |
| 7 | de novo | Gain | 3q29 | 1653 | 29 | 863 | 1 | 8.33 x 10^−3^ | rs338217 | DLG1 promoter |
| 8 | maternal | Loss | 1q21.1 | 2116 | 26 | 975 | 1 | 1.42 x 10^−6^ | rs2120003 | FMO5 |
| 9 | maternal | Loss | 22q11.22 | 259 | 1 | 437 | 9.95 x 10^−1^ | 4.29 x 10^−2^ | rs6000510 | TOP3B, VPREB1 |
| 10 | maternal | Gain | 17q11.2 | 393 | 2 | 279 | 9.86 x 10^−1^ | 5.57 x 10^−2^ | rs2466848 | MYO1D, TMEM98 |
| 11 | maternal | Gain | 5q22.1-q23.2 | 14229 | 59 | 15390 | 1 | 2.08 x 10^−8*^ | rs1582931 | CEP120 |
| 12 | maternal | Gain | 1q21.1 | 307 | 6 | 49 | 1 | 5.37 x 10^−1^ | rs1297552 | CD160, PDZK1 |
| 13 | maternal | Gain | 2q33.2 | 323 | 3 | 93 | 1 | 1.01 x 10^−4^ | rs3731696 | BMPR2 3‘ UTR |
| 14 | maternal | Gain | 7q36.3 | 165 | 1 | 125 | 9.83 x 10^−1^ | 1.13 x 10^−2^ | rs1182443 | UBE3C |
| 15 | maternal | Gain | 1p36.33 | 306 | 20 | 79 | 9.54 x 10^−1^ | 5.25 x 10^−2^ | rs4075116 | AGRN, RNF223 |
| 16 | maternal | Gain | 2q21.2 | 598 | 3 | 694 | 1 | 8.76 x 10^−4^ | rs4953859 | NCKAP5 |
| 17 | paternal | Loss | 13q22.1 | 118 | 1 | 67 | 6.92 x 10^−1^ | 1.43 x 10^−2^ | rs17211014 | PIBF1 |
| 18 | paternal | Loss | 14q21.1-q21.2 | 1871 | 1 | 2036 | 1 | 4.32 x 10^−2^ | rs1952915 | LRFN5 |
| 19 | paternal | Loss | 1q21.1 | 1654 | 17 | 983 | 1 | 1.42 x 10^−6^ | rs2120003 | FMO5 |
| 20 | paternal | Loss | 5p15.33 | 109 | 1 | 25 | 9.91 x 10^−1^ | 1.11 x 10^−1^ | rs1709544 | TPPP |
