# Supplementary material for: Rare Copy Number Variants Are a Common Cause of Short Stature
Source: PLoS Genet. 2013 Mar 14;9(3):e1003365. doi: 10.1371/journal.pgen.1003365 (PMC3597495; doi:10.1371/journal.pgen.1003365)
Supplement: Table S6 — Significant differentially expressed candidate genes within the identified CNVs. (DOCX) [file pgen.1003365.s010.docx]

| **Table S6. Significant differentially expressed candidate genes within the identified CNVs** | | | | | | |
| --- | --- | --- | --- | --- | --- | --- |
| Gene Symbol | Position | Type | *P* value | Ratio | Fold change | Patient |
| DOWN REGULATED | |  |  |  |  |  |
| PPP1R15B | 1q32.1 | Deletion | 3.6 X 10^-4^ | 0.45 | -2.20 | 1 |
| PIK3C2B | 1q32.1 | Deletion | 5.9 X 10^-3^ | 0.32 | -3.13 | 1 |
| MDM4 | 1q32.1 | Deletion | 1.5 X 10^-2^ | 0.38 | -2.65 | 1 |
| RBBP5 | 1q32.1 | Deletion | 4.4 X 10^-2^ | 0.47 | -2.12 | 1 |
| DSTYK | 1q32.1 | Deletion | 1.7 X 10^-3^ | 0.42 | -2.40 | 1 |
| NUAK2 | 1q32.1 | Deletion | 2.0 X 10^-4^ | 0.26 | -3.84 | 1 |
| NUCKS1 | 1q32.1 | Deletion | 2.3 X 10^-3^ | 0.49 | -2.06 | 1 |
| RAB7L1 | 1q32.1 | Deletion | 2.0 X 10^-3^ | 0.53 | -1.89 | 1 |
| SLC41A1 | 1q32.1 | Deletion | 6.7 X 10^-5^ | 0.32 | -3.08 | 1 |
| EPHA4 | 2q36.1-36.3 | Deletion | 4.4 X 10^-2^ | 0.39 | -2.59 | 2 |
| ACSL3 | 2q36.1-36.3 | Deletion | 2.6 X 10^-3^ | 0.49 | -2.03 | 2 |
| WDFY1 | 2q36.1-36.3 | Deletion | 2.2 X 10^-2^ | 0.31 | -3.23 | 2 |
| MRPL44 | 2q36.1-36.3 | Deletion | 6.1 X 10^-3^ | 0.52 | -1.91 | 2 |
| CUL3 | 2q36.1-36.3 | Deletion | 2.1 X 10^-2^ | 0.49 | -2.05 | 2 |
| DOCK10 | 2q36.1-36.3 | Deletion | 7.4 X 10^-3^ | 0.51 | -1.95 | 2 |
| AGFG1 | 2q36.1-36.3 | Deletion | 1.8 X 10^-3^ | 0.42 | -2.37 | 2 |
| EXOC5 | 14q23.1 | Deletion | 4.3 X 10^-2^ | 0.70 | -1.44 | 3 |
| MUDENG | 14q23.1 | Deletion | 1.8 X 10^-3^ | 0.50 | -2.01 | 3 |
| FOSL2 | 2p23.3 | Duplication | 1.04 X 10^-3^ | 0.45 | -2.22 | 5 |
| PRKAB2 | 1q21.1 | Deletion | 4.3 X 10^-3^ | 0.60 | -1.67 | 19 |
| ACP6 | 1q21.1 | Deletion | 3.2 X 10^-4^ | 0.70 | -1.42 | 19 |
| UP REGULATED | |  |  |  |  |  |
| FAM72 | 1q32.1 | Deletion | 1.49 X 10^-2^ | 2.21 | 2.21 | 1 |
| DNMT3A | 2p23.3 | Duplication | 7.8 X 10^-3^ | 1.31 | 1.31 | 5 |
| HADHA | 2p23.3 | Duplication | 8.4 X 10^-3^ | 1.28 | 1.28 | 5 |
| HADHB | 2p23.3 | Duplication | 5.1 X 10^-3^ | 1.32 | 1.32 | 5 |
| X 10PT1 | 2p23.3 | Duplication | 1.9 X 10^-4^ | 1.36 | 1.36 | 5 |
| TM X 10M214 | 2p23.3 | Duplication | 4.4 X 10^-3^ | 1.47 | 1.47 | 5 |
| AGBL5 | 2p23.3 | Duplication | 3.4 X 10^-5^ | 1.30 | 1.30 | 5 |
| OST4 | 2p23.3 | Duplication | 3.0 X 10^-2^ | 1.79 | 1.79 | 5 |
| KHK | 2p23.3 | Duplication | 6.4 X 10^-7^ | 1.40 | 1.40 | 5 |
| PR X 10B | 2p23.3 | Duplication | 8.2 X 10^-3^ | 1.67 | 1.67 | 5 |
| SLC5A6 | 2p23.3 | Duplication | 1.2 X 10^-2^ | 1.76 | 1.76 | 5 |
| C2orf28 | 2p23.3 | Duplication | 1.4 X 10^-3^ | 2.05 | 2.05 | 5 |
| CAD | 2p23.3 | Duplication | 6.4 X 10^-3^ | 2.02 | 2.02 | 5 |
| MPV17 | 2p23.3 | Duplication | 1.4 X 10^-3^ | 2.60 | 2.60 | 5 |
| GTF3C2 | 2p23.3 | Duplication | 4.2 X 10^-3^ | 2.07 | 2.07 | 5 |
| X 10IF2B4 | 2p23.3 | Duplication | 1.6 X 10^-2^ | 3.61 | 3.61 | 5 |
| SNX17 | 2p23.3 | Duplication | 1.0 X 10^-2^ | 1.70 | 1.70 | 5 |
| PPM1G | 2p23.3 | Duplication | 8.0 X 10^-4^ | 2.00 | 2.00 | 5 |
| ZNF512 | 2p23.3 | Duplication | 6.2 X 10^-4^ | 2.10 | 2.10 | 5 |
| GPN1 | 2p23.3 | Duplication | 4.2 X 10^-3^ | 2.06 | 2.06 | 5 |
| SUPT7L | 2p23.3 | Duplication | 6.2 X 10^-5^ | 1.72 | 1.72 | 5 |
| MRPL33 | 2p23.3 | Duplication | 8.2 X 10^-3^ | 1.42 | 1.42 | 5 |
| BR X 10 | 2p23.3 | Duplication | 1.8 X 10^-3^ | 1.36 | 1.36 | 5 |
| TRMT61B | 2p23.3 | Duplication | 2.4 X 10^-3^ | 2.38 | 2.38 | 5 |
| ZNF256 | 19q13.43 | Duplication | 8.9 X 10^-3^ | 1.96 | 1.96 | 6 |
| TFRC | 3q29 | Duplication | 5.6 X 10^-3^ | 2.02 | 2.02 | 7 |
| PCYT1A | 3q29 | Duplication | 1.7 X 10^-2^ | 1.40 | 1.40 | 7 |
| TCT X 10X1D2 | 3q29 | Duplication | 3.5 X 10^-6^ | 4.00 | 4.00 | 7 |
| WDR53 | 3q29 | Duplication | 1.1 X 10^-3^ | 2.62 | 2.62 | 7 |
| FBXO45 | 3q29 | Duplication | 2.1 X 10^-4^ | 2.43 | 2.43 | 7 |
| LRRC33 | 3q29 | Duplication | 3.2 X 10^-2^ | 1.66 | 1.66 | 7 |
| S X 10NP5 | 3q29 | Duplication | 7.5 X 10^-3^ | 1.29 | 1.29 | 7 |
| NCBP2 | 3q29 | Duplication | 1.2 X 10^-3^ | 2.60 | 2.60 | 7 |
| LOC152217 | 3q29 | Duplication | 2.3 X 10^-3^ | 3.41 | 3.41 | 7 |
| DLG1 | 3q29 | Duplication | 2.1 X 10^-4^ | 4.75 | 5.75 | 7 |
| BDH1 | 3q29 | Duplication | 4.3 X 10^-4^ | 4.07 | 4.07 | 7 |
| CD160 | 1q21.1 | Duplication | 3.3 X 10^-2^ | 3.86 | 3.86 | 12 |
| GPR89A | 1q21.1 | Duplication | 5.3 X 10^-3^ | 2.05 | 2.05 | 12 |
| GPR89C | 1q21.1 | Duplication | 6.9 X 10^-3^ | 2.06 | 2.06 | 12 |
