# Supplementary material for: Rare Copy Number Variants Are a Common Cause of Short Stature
Source: PLoS Genet. 2013 Mar 14;9(3):e1003365. doi: 10.1371/journal.pgen.1003365 (PMC3597495; doi:10.1371/journal.pgen.1003365)
Supplement: Table S7 — Network analysis of affected genes within the identified candidate CNVs. (DOCX) [file pgen.1003365.s011.docx]

| **Table S7. Network analysis of affected genes within identified candidate CNVs** | | |
| --- | --- | --- |
| ID | Associated Network Functions | Score |
| ALL AFFECTED GENES | | |
| 1 | Developmental Disorder, Hereditary Disorder, Metabolic Disease | 43 |
| 2 | Cell Morphology, Nervous System Development and Function, Tissue Morphology | 31 |
| 3 | Cellular Function and Maintenance, Hematological System Development and Function, Cell-To-Cell Signaling and Interaction | 31 |
| 4 | Reproductive System Disease, Cell Morphology, Skeletal and Muscular System Development and Function | 27 |
| 5 | Dermatological Diseases and Conditions, Developmental Disorder, Hereditary Disorder | 27 |
| SIGNIFICANT DIFFERENTIALLY EXPRESSED GENES | | |
| 1 | Developmental Disorder, Genetic Disorder, Metabolic Disease | 39 |
| 2 | Neurological Disease, Genetic Disorder, Nutritional Disease | 31 |
| 3 | Cell Death, DNA Replication, Recombination, and Repair, Free Radical Scavenging | 28 |
| 4 | Cellular Movement, Cell Cycle, Cell Signaling | 23 |
| 5 | Cell Death, Cellular Growth and Proliferation, Cellular Movement | 5 |
| SIGNIFICANT DIFFERENTIALLY EXPRESSED GENES – DELETED ONLY | | |
| 1 | Cellular Assembly and Organization, Nervous System Development and Function, Tissue Development | 37 |
| 2 | Cell Cycle, Cell Death, Protein Degradation | 13 |
| SIGNIFICANT DIFFERENTIALLY EXPRESSED GENES – DUPLICATED ONLY | | |
| 1 | Cell Cycle, Carbohydrate Metabolism, Lipid Metabolism | 34 |
| 2 | Cellular Compromise, Cell Morphology, Cellular Assembly and Organization | 28 |
| 3 | Developmental Disorder, Genetic Disorder, Metabolic Disease | 19 |
| 4 | Cellular Assembly and Organization, Increased Levels of Alkaline Phosphatase, Connective Tissue Development and Function | 3 |
| 5 | Gene Expression | 3 |
| The calculated score is based on the hypergeometric distribution with the right-tailed Fisher's Exact Test. The score presents the negative log of the p value. | | |
